# The optimal course and frequency of Tai Chi for knee osteoarthritis: a systematic review and meta-analysis of randomized controlled trials

**DOI:** 10.3389/fpubh.2025.1661674

**Published:** 2025-10-28

**Authors:** Jing Deng, Leyi Zhang, Fengjiao Chen, Yufeng Tao, Hao Yang, Lanlan Yu, Chi Zhang

**Affiliations:** ^1^School of Health Preservation and Rehabilitation, Chengdu University of Traditional Chinese Medicine, Chengdu, Sichuan, China; ^2^School of Clinical Medicine, Chengdu University of Traditional Chinese Medicine, Chengdu, Sichuan, China

**Keywords:** Tai Chi, knee osteoarthritis, optimal course and frequency, systematic review, meta-analysis

## Abstract

**Objectives:**

Knee osteoarthritis (KOA) is a highly prevalent degenerative joint disease worldwide and an important cause of disability. Currently, medication and surgical interventions are commonly used in clinical practice, but there are limitations such as significant side effects and high medical costs. Tai Chi, as a non-pharmacologic intervention, is recommended for its safety and few adverse effects. However, there is still a lack of consensus on the optimal course and frequency of Tai Chi intervention, and there is an urgent need to optimize clinical intervention protocols. In order to scientifically assess the optimal course and frequency of Tai Chi for the treatment of KOA, this study integrates the existing evidence through a systematic review and meta-analysis, and aims to provide standardized protocols for Tai Chi training in clinical practice.

**Methods:**

PubMed, Embase, Cochrane Library, Web of Science, Scopus, EBSCO, CNKI, Wanfang Database, and VIP database were searched from establishment to August 30, 2025. Two reviewers independently extracted data and assessed the quality of the literature and the certainty of the evidence for each outcome according to the Cochrane Risk of Bias Tool and the Grading of Recommendations, Assessment, Development & Evaluation (GRADE) approach. Outcome measures included Western Ontario and McMaster Universities Osteoarthritis Index (WOMAC) pain, WOMAC stiffness, WOMAC physical function, Visual Analogue Scale (VAS) pain, 36-item Short Form Health Survey (SF-36) Physical Component Summary (PCS), and SF-36 Mental Component Summary (MCS). For combined outcomes, standardized mean difference (SMD) and 95% confidence interval (CI) were calculated. Review Manager 5.4.1, Stata 15.0 and GRADE profiler software were used to statistically analyze and plot the included information.

**Results:**

A total of 13 randomized controlled trial (RCT) studies (*n* = 701) were included in this review. The results of the meta-analysis showed that Tai Chi relieved pain (WOMAC pain: SMD = −0.41, 95%CI [−0.58, −0.25], *p* < 0.01; VAS pain: SMD = −0.33, 95% CI [−0.57, −0.10], *p* < 0.01), reduced stiffness (SMD = −0.27, 95% CI [−0.43, −0.11], *p* < 0.01), improved physical function (SMD = −0.52, 95% CI [−0.68, −0.36], *p* < 0.01), and improved physical health (SMD = 0.47, 95% CI [0.27, 0.67], *p* < 0.01). Subgroup analyses showed that the long-term (>16 weeks)/three-times-weekly Tai Chi training protocol was optimal (SMD = −0.74, 95% CI [−1.06, −0.41], *p* < 0.01; SMD = −0.96, 95% CI [−1.30, −0.63], *p* < 0.01) in terms of improvement of pain and physical function; and that in terms of improvement of stiffness, the short-term (≤16 weeks)/three-times-weekly Tai Chi training protocol was optimal (SMD = −0.52; 95% CI [−0.84, −0.19], *p* < 0.01); and in terms of improving physical functioning, a short-term (≤16 weeks)/twice-weekly Tai Chi training protocol was optimal (SMD = 0.44, 95% CI [0.21, 0.68], *p* < 0.01).

**Conclusion:**

This meta-analysis suggests that Tai Chi is effective in improving pain, stiffness, physical function, and physical health in patients with KOA. Patients with KOA should consider their specific conditions and choose a Tai Chi training protocol that suits their needs. The preliminary results of this meta-analysis indicate that for patients with pain and physical functional limitations, a long-term (>16 weeks)/three times weekly Tai Chi training regimen may be selected; for patients experiencing knee stiffness, a short-term (≤16 weeks)/three times weekly Tai Chi training regimen may be considered; and for KOA patients seeking to improve physical health through Tai Chi training, a short-term (≤16 weeks)/twice weekly Tai Chi training regimen may be selected. However, the number of large-sample studies in this review is limited, and more studies are urgently needed to confirm these results.

**Systematic review registration:**

Identifier–CRD42024599921, https://www.crd.york.ac.uk/PROSPERO/myprospero.

## 1 Introduction

Knee osteoarthritis (KOA) is a degenerative knee disease that can lead to pain, functional limitations, disability, and psychological disorders, and reduce the overall quality of life of patients. Globally, KOA impacts over 260 million people, making it one of the leading causes of disability, as reported in the 2021 Global Burden of Disease Study (1, 2) prevalence, polypharmacy and number of drug prescriptions confirm the increased burden of KOA (3). Therefore, finding effective means to improve pain, limitation of movement, and quality of life in older adults with KOA has become a critical issue requiring urgent clinical attention.

Currently, KOA is treated with various means, which are mainly categorized into surgical treatment, pharmacological treatment and non-pharmacological treatment. Although surgical treatment is effective in relieving the symptoms of KOA, it is usually considered only after conservative treatment fails, given the higher risks and complications of surgery in the older adults (4). Pharmacologic treatments, such as non-steroidal anti-inflammatory drugs, can relieve KOA symptoms. However, their long-term use is associated with adverse side effects, including gastrointestinal complications and cardiovascular risks (5, 6). Treatment of patients with KOA should be comprehensive, and non-pharmacologic therapy is considered the primary treatment (7). The American College of Rheumatology (ACR) strongly recommends Tai Chi as an effective and safe complementary and alternative approach to KOA management to regulate the mind and body (6, 8).

Tai Chi, a traditional Chinese mind–body exercise, combines breath regulation, gentle movements, and meditation, focusing on physical and mental balance. A growing number of RCTs and meta-analyses have demonstrated its efficacy in relieving pain, improving physical function and enhancing mental health in patients with KOA (9, 10). However, improper practice can lead to knee injuries. Therefore, it is especially important to determine the optimal course and frequency of sessions in order to maximize the therapeutic benefits of Tai Chi and avoid potential injuries.

Published literature suggests that the course and frequency settings for Tai Chi treatment of KOA vary widely. Some studies used short -term courses, such as 6 or 12 weeks, while others used longer -term courses, such as 24 or 36 weeks. In terms of frequency, Tai Chi practice in different studies ranged from twice-weekly to three-times-weekly. Previous systematic reviews have examined the effects of Tai Chi training on KOA, but have not yet focused on the role of course and frequency on efficacy. In order to further optimize the Tai Chi treatment protocol for KOA, this study deeply investigated the effects of three different course and frequency combinations [short-term (≤16 weeks)/twice-weekly; short-term (≤16 weeks)/three-times-weekly; and long-term (>16 weeks)/three-times-weekly] on the symptoms of KOA through subgroup analyses with the aim of providing a scientific and reasonable Tai Chi training protocol in the clinic.

## 2 Methods

This systematic review and meta-analysis was reported according to the Preferred Reporting Items for Systematic Reviews and Meta-Analyses (PRISMA) guidelines (11). The review panel conducted the systematic review and meta-analysis in accordance with the PRISMA checklist (Supplementary Table S1). This protocol was registered in the Prospective Register of Systematic Reviews (http://www.crd.york.ac.uk/PROSPERO, ID: CRD42024599921) before the review was conducted.

### 2.1 Search strategy

A literature search was conducted by two independent reviewers (J.D. and Y.T.). Articles were retrieved from nine electronic databases: PubMed, Embase, Cochrane Library, Web of Science, Scopus, EBSCO, CNKI, Wanfang Database, and VIP database. We included papers published from database inception to August 30, 2025 in English and Chinese. MeSH Terms included synonyms of “Knee Osteoarthritis,” “Tai Chi,”” randomized controlled trial” and their combinations. A more detailed search strategy is in Supplementary Table S2.

### 2.2 Inclusion and exclusion criteria

Articles were included for this research based on the following criteria: (1) the included studies were all RCTs published in English and Chinese; (2) participants were diagnosed with KOA by validated criteria, such as the ACR, the Kellgren Lawrence classification (KL), Diagnosis and Treatment Guidelines for Osteoarthritis 2010 issued by the Chinese Medical Association, radio-graphic evidence or physician-confirmed diagnosis, regardless of age, race or gender; (3) outcome measures WOMAC pain, WOMAC stiffness, WOMAC physical function, VAS pain, SF-36 PCS, and SF-36 MCS were assessed; (4) the intervention group was treated with Tai Chi as the only treatment, with no restriction on the type of Tai Chi, while the control group received other treatments besides Tai Chi, such as attention control, health education, physical therapy, balance and postural control training or no intervention.

Articles were excluded based on the following criteria: (1) not RCT; (2) duplicate data; (3) full text was not retrieved, and (4) the patients with mental disorders such as mild cognitive impairment and dementia, and after knee replacement surgery.

### 2.3 Study selection

The retrieved articles were imported into the literature management software EndNote 21 to eliminate duplicate articles. Two reviewers (J.D. and L.Z.) independently read the abstracts and titles of these articles according to the inclusion and exclusion criteria described above and excluded articles that were inconsistent with this study. Then, the potentially eligible articles were further read in full text for assessment. Discrepancies were resolved by the third reviewer (F.C.).

### 2.4 Data extraction

Two reviewers (J.D. and L.Z.) independently extracted key information from the final screened articles and imported them into Microsoft Excel 2021. Key information extracted includes: first author, publication year, country, age of participants, diagnostic criteria, sample size, intervention measures (intervention group and control group), intervention course and frequency, outcome indicators (WOMAC/VAS/SF-36), adverse effects. If missing information is encountered, we will email the first author to inquire about it, and the study will be abandoned if there is no response twice. After the information had been fully extracted, the two reviewers performed a cross-check. If there were disagreements between the two reviewers, discussions were held to resolve those disagreements, and a third reviewer (F.C.) provided recommendations to ensure the accuracy of the information when the disagreements could still not be resolved.

### 2.5 Risk of bias assessment and GRADE

Two reviewers (J.D. and L.Z.) independently evaluated the risk of bias of included studies by using Cochrane Risk of Bias Tool (12). The assessed items included random sequence generation, allocation sequence concealment, blinding of subjects and investigators, blinding of outcome measurers, outcome data incompleteness, selective reporting, and other potential sources of bias. Each study was evaluated as “low risk of bias,” “high risk of bias” or “unclear” according to the risk of bias assessment criteria. The quality of evidence rating of the results of the subgroup analyses, including the risk of bias, inconsistency, indirectness, imprecision, and publication bias, were categorized as very low, low, moderate, or high judgment using the GRADE profiler software. Any disagreements between the two reviewers were decided through consultation with the third reviewer (F.C.).

### 2.6 Assessment of reporting quality

Two reviewers (J.D. and L.Z.) independently used PRISMA 2020 to assess the quality of the reports. The PRISMA 2020 statement, which contains 27 items, evaluates the quality of the reports of the included literature in seven aspects: title, abstract, background, methods, results, discussion, and other information. If there were disagreements, they were discussed with the third reviewer (F.C.) to reach a consensus.

### 2.7 Statistical analysis

The statistical software Review Manager 5.4.1 was used in this study for study quality evaluation, data merging, heterogeneity testing, and forest plot generation for the included studies. The outcomes of this study were WOMAC pain, WOMAC stiffness, WOMAC physical function, VAS pain, SF-36 PCS, and SF-36 MCS. We extracted quantitative data from all selected RCTs, including sample sizes as well as the mean and standard deviation (SD) of values measured for each group at baseline and post-intervention. Raw data and calculation of mean and SD for all RCTs are shown in Supplementary Table S3. Considering that all variables included in the studies were reported as continuous data, we used SMD with 95%CI to estimate effect sizes. Clinically, effect sizes were categorized as small (<0.40), moderate (0.40–0.70), or large (>0.70) based on the SMD (13). We considered *p* < 0.05 as statistically significant.

Heterogeneity between studies was assessed by *I*^2^ values: *I*^2^ ≤ 25%, low heterogeneity; 25% < *I*^2^ < 50%, moderate heterogeneity; 50% < *I*^2^ < 75%, substantial heterogeneity; *I*^2^ ≥ 75%, high heterogeneity (14). When *I*^2^ < 50% and *p* ≥ 0.1, a fixed-effects model was used for meta-analysis; otherwise, a random-effects model was used for analysis (9). When the heterogeneity was substantial, subgroup analysis, sensitivity analysis and Egger’s test were performed with Review Manager 5.4.1 and Stata 15.0 to determine whether the results of meta-analysis are stable and to examine the potential bias in the RCTs included in this meta-analysis.

Subgroup analyses were used to explore the optimal course and frequency of Tai Chi training, including three protocols: short-term (≤16 weeks)/twice-weekly; short-term (≤16 weeks)/three-times-weekly; and long-term (>16 weeks)/three-times-weekly. Although the American College of Rheumatology guidelines emphasize the need for regular, sustained exercise interventions to maintain therapeutic outcomes in patients with osteoarthritis (15), they do not clearly define the limits between short-term and long-term interventions. Based on the actual allocation of included studies, we selected the mean intervention course (16 weeks) as the threshold for distinguishing between short-term and long-term protocols.

## 3 Results

### 3.1 Search results

**Figure 1:**
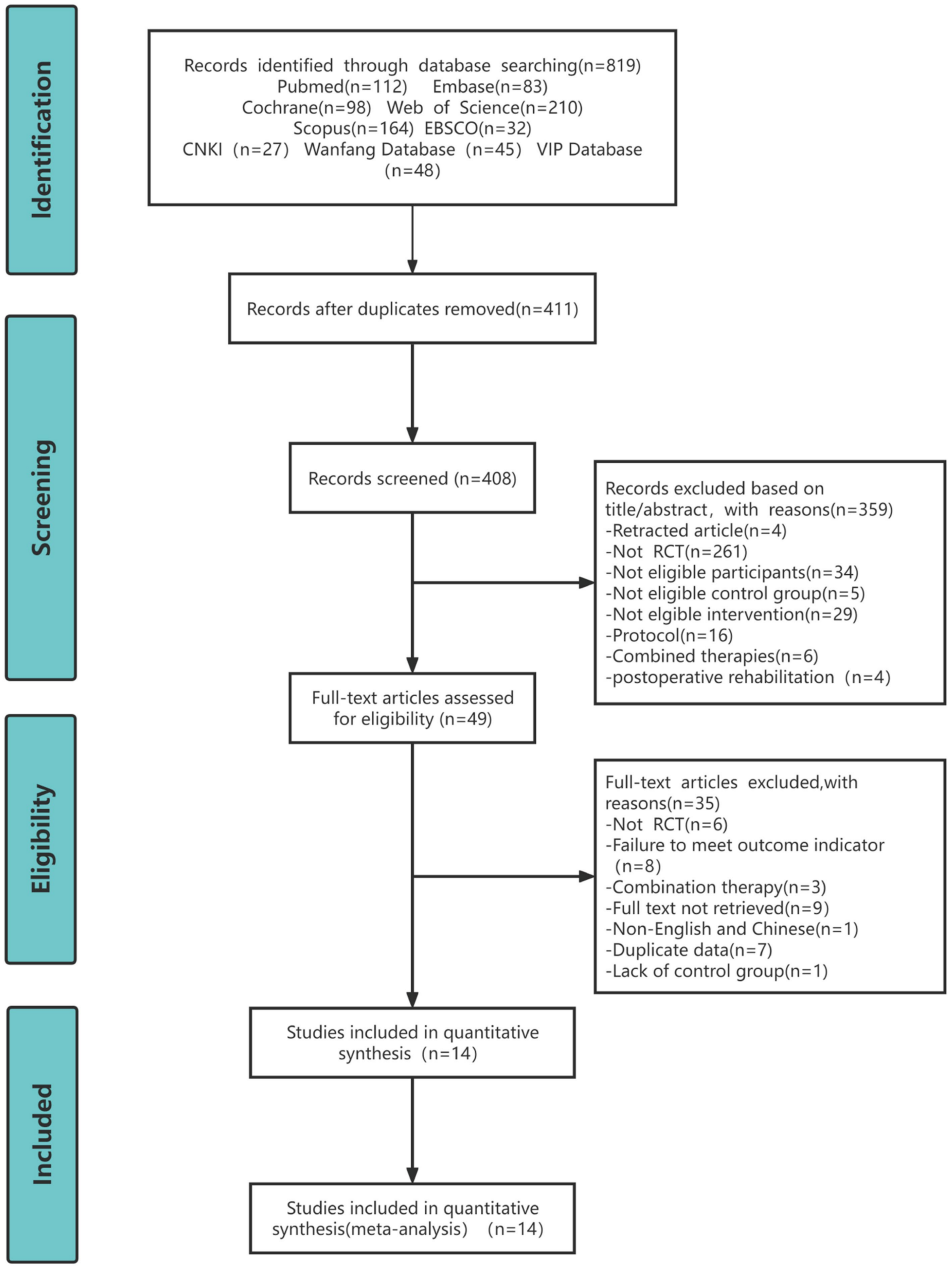
PRISMA flow chart for study screening.

The research screening process is shown in Figure 1, and details of the excluded studies and reasons for exclusion are provided in Supplementary Table S4. A total of 819 articles were identified through a search of nine different electronic databases. The bibliography was imported into EndNote 21 and 408 articles remained after excluding duplicates. 359 studies that did not meet the inclusion criteria for this systematic review were excluded by title and abstract. Then, the full text of the remaining 49 articles was evaluated further and 35 studies were excluded for the following reasons: non-RCT (*n* = 6); duplicate data (*n* = 7); full text cannot be retrieved (*n* = 9); failure to meet outcome indicators (*n* = 8); combination therapy (*n* = 3); and non-Chinese English (*n* = 1). Finally, a total of 14 studies were included in this meta-analysis, of which two articles were the same study with different outcome indicators (Lü’s study used SF-36 as the outcome measure, while Zhu’s study employed WOMAC; both SF-36 and WOMAC are required measures for this meta-analysis), so 13 RCTs were finally included (16, 17).

### 3.2 Study characteristics

Basic characteristics of the included studies are shown in Table 1. A total of 13 RCTs containing 701 participants were included in this study, all of which were published before August 30, 2025 in English and Chinese. The studies were conducted, respectively, in China (*n* = 7) (10, 16–21), the United States (*n* = 4) (22–25), and South Korea (*n* = 2) (26, 27). Participants were diagnosed with KOA by ACR, KL, Diagnosis and Treatment Guidelines for Osteoarthritis 2010 issued by the Chinese Medical Association, radio-graphic evidence, or physician-confirmed.

**Table 1:** Basic characteristics of the included studies.

Author, year	Country	Patients diagnostic criteria	Mean age (year) ± SD	Sample size (IG/CG)	Intervention group	Control group	Outcomes measure	Adverse effects
彭 and 唐, 2021 (18)	China	DTGOA- 2010(CMA)	IG:63.0 ± 5.1 CG:59.0 ± 6.9	20/20	Tai Chi 6 weeks/3 times weekly	Oral Chinese medicine 6 weeks/1 dose/day	WOMAC Pain/Stiffness/Physical function VAS Pain	No adverse event
Wang et al. 2009 (25)	USA	ACR	IG:63 ± 8.1 CG:68 ± 7.0	20/20	Tai Chi (Yang style) 12 weeks/twice weekly	Attention control 12 weeks/twice weekly	WOMAC Pain/Stiffness/Physical function VAS pain SF-36(PCS/MCS)	One participant in the Tai Chi group reported an increase in knee pain
周, 2019 (19)	China	Physician-confirmed diagnosis	IG:64.08 ± 1.05 CG:64.21 ± 0.98	15/15	Tai Chi 16 weeks/twice weekly	None	VAS pain	No adverse event
Wang et al. 2016 (22)	USA	ACR	IG:60.3 ± 10.5 CG:60.1 ± 10.5	106/98	Tai Chi (Yang style) 12 weeks/twice weekly	Physical therapy Twice weekly for the first 6 weeks and 4 times weekly for the second 6 weeks	WOMAC Pain/Stiffness/Physical function SF-36(PCS/MCS)	No adverse event
Kang et al. 2022 (20)	China	Physician-confirmed diagnosis	IG:63.4 ± 4.6 CG:64.7 ± 6.1	12/15	Tai Chi (Chen style) 36 weeks/3 times weekly	Wellness education 36 weeks/once a month	WOMAC Pain/Stiffness/Physical function SF-36(PCS/MCS)	No adverse event
Lü et al. 2017 (16)	China	ACR	IG:64.61 ± 3.40 CG:64.53 ± 3.43	21/19	Tai Chi 24 weeks/3 times weekly	Health education 24 weeks/bi-weekly	SF-36(PCS/MCS)	No adverse event
Zhu et al. 2016 (17)							WOMAC Pain/Stiffness/Physical function	No adverse event
Brisme’e et al. 2007 (23)	USA	ACR	IG:70.8 ± 9.8 CG:68.89 ± 8.9	18/13	Tai Chi (Yang style) 12 weeks/3 times weekly	Attention control 6 weeks/3 times weekly	WOMAC Pain/Stiffness/Physical function	No adverse event
Song et al. 2003 (26)	Korea	ACR	IG:64.8 ± 6.0 CG:62.5 ± 5.6	22/21	Tai Chi (Sun style) 12 weeks/3 times weekly	None	WOMAC Pain/Stiffness/Physical function	No adverse event
Hu et al. 2019 (21)	China	Radiographic evidence	IG:66.32 ± 4.16 CG:65.54 ± 3.59	52/40	Tai Chi 24 weeks/3 times weekly	Health education 24 weeks/not mentioned	WOMAC Pain/Stiffness/Physical function VAS Pain	No adverse event
Song et al. 2022 (10)	China	Physician-confirmed diagnosis	IG:64.15 ± 8.56 CG:64.15 ± 8.56	20/20	Tai Chi (Yang style) 12 weeks/3 times weekly	Health education 12 weeks/once a week	WOMAC Pain/Stiffness/Physical function SF-36(PCS/MCS)	No adverse event
Wortley et al. 2013 (24)	USA	ACR	IG:68.1 ± 5.3 CG:70.5 ± 5.0	12/6	Tai Chi (Yang style) 10 weeks/twice weekly	None	WOMAC Pain/Stiffness/Physical function	No adverse event
Lee et al. 2009 (27)	Korea	KL scale	IG: 70.2 ± 4.8 CG:66.9 ± 6.0	29/15	Tai Chi 8 weeks/twice weekly	None	WOMAC Pain/Stiffness/Physical function SF-36(PCS/MCS)	No adverse event
Zhang et al. 2025 (28)	China	ACR	IG:60.58 ± 5.66 CG:58.21 ± 5.18	24/28	Tai Chi (Yang style) 12 weeks/twice weekly	Balance and postural control training 12 weeks/twice weekly	VAS pain	No adverse event

ACR, American College of Rheumatology; IG, Intervention Group; CG, Control Group; KL scale, Kellgren–Lawrence Scale; WOMAC, Western Ontario and McMaster Universities Osteoarthritis Index; DTGOA - 2010 (CMA), Diagnosis and Treatment Guidelines for Osteoarthritis 2010 issued by the Chinese Medical Association; SF-36, the 36-item Short Form Health Survey; PCS, Physical Component Summary; MCS, Mental Component Summary; VAS, Visual Analogue Scale.

In terms of intervention protocols, the intervention group was not restricted in the type of Tai Chi and the intervention course ranged from 6 to 36 weeks. Based on training course and frequency, we categorized the 13 studies into three protocols: short-term (≤16 weeks)/twice-weekly (10, 19, 22, 24, 25, 27, 28); short-term (≤16 weeks)/three-times-weekly (10, 18, 22, 23, 26); and long-term (>16 weeks)/three-times-weekly (16, 17, 20, 21). The control group in 2 studies received attention control (23, 25), 4 studies received health education (10, 16, 17, 20–22), 1 study received physical therapy (22), 1 study received herbal medicine (18), 1 study received balance and postural control training (28), and 4 studies had no intervention (19, 24, 26, 27).

Eleven studies used WOMAC as an outcome indicator (16–18, 20–27), 6 studies used VAS (18, 19, 21, 23, 25, 28), and 7 studies used SF-36 (10, 16, 17, 20, 22, 22, 25, 27), WOMAC scores were reported differently across studies, with 9 reporting 0–100 (10, 16–22, 23, 26, 27), and 3 reporting 0–1,700 (10, 22, 24, 25). Higher scores on WOMAC and VAS reflected poorer conditions, whereas higher scores on SF-36 reflected better quality of life. Six studies chose the Yang-style Tai Chi (10, 22–25, 28), 1 study chose the Chen-style Tai Chi (20), 1 study chose the Sun-style Tai Chi (26), and the remaining 5 studies did not mention the style of Tai Chi. Finally, only one study reported adverse events and the remaining studies did not report any adverse events (25).

### 3.3 Assessment of risk of bias

**Figure 2:**
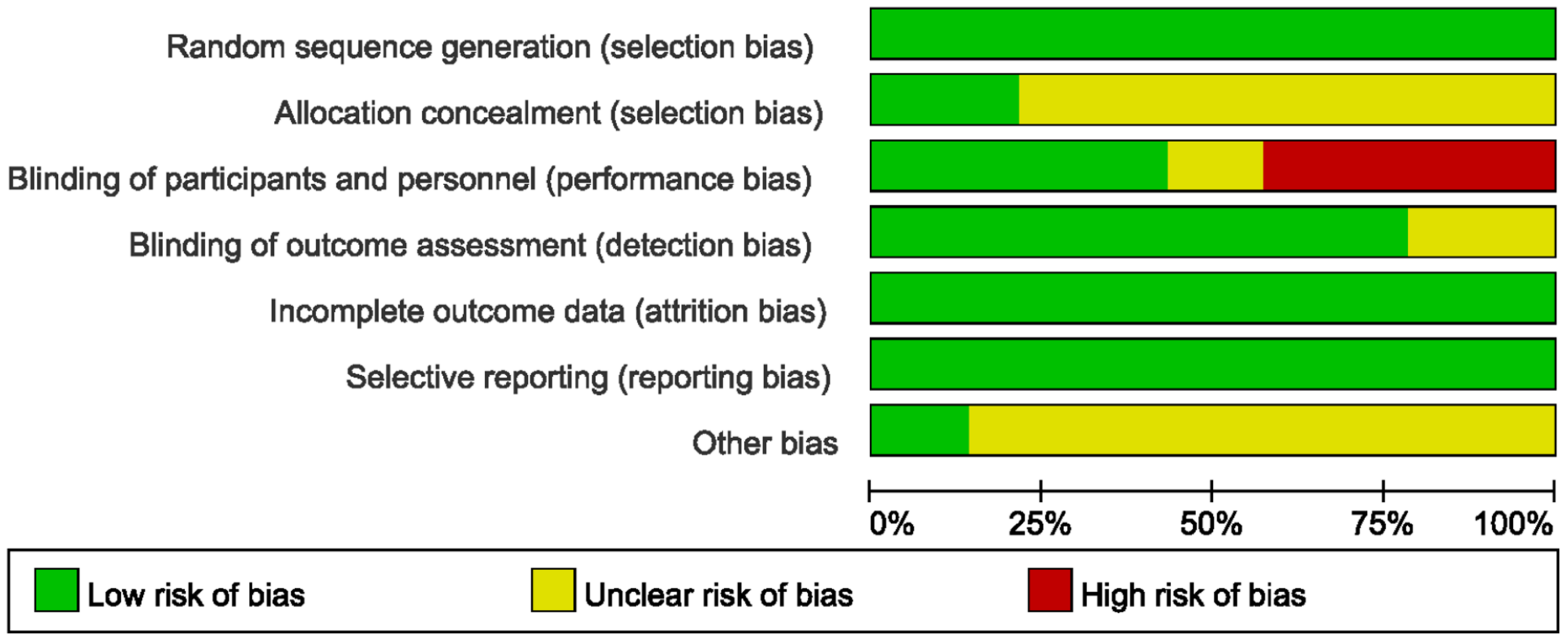
Risk of bias graph.

**Figure 3:**
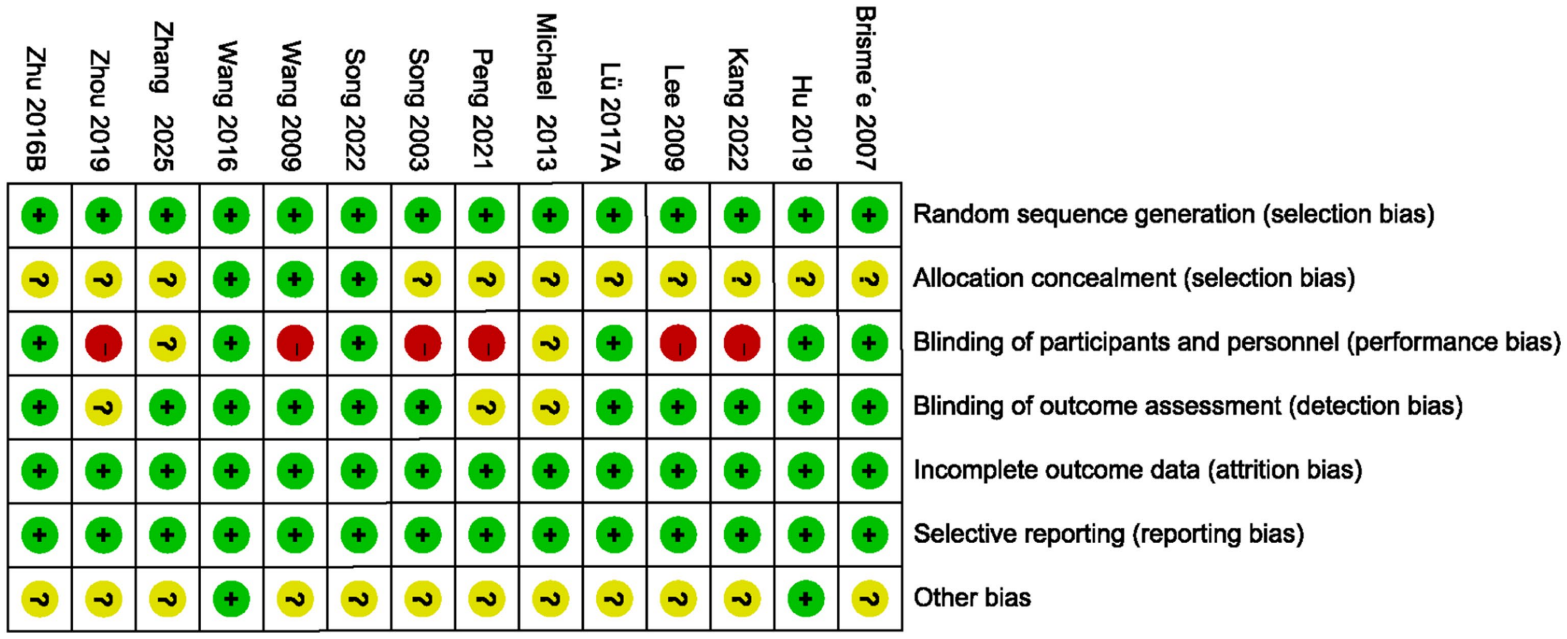
Risk of bias summary.

The risk of bias assessment results for all included studies are summarized in Figure 2, 3, and detailed reasons for bias are provided in Supplementary Table S5. Of the 13 included studies, all described the process of generating randomized sequences, which was considered low risk. Among these studies, 3 studies (10, 22, 22, 25) reported allocation concealment and 5 (10, 16, 17, 21–23) reported blinding of participants and researchers. For the completion of outcome data and assessment of blinding, 13 and 10 (10, 16, 17, 20–23, 25–28) included studies presented low risk, respectively. Thirteen studies reported the number of lost visits and the reasons for them. Finally, 11 articles (10, 16–20, 22, 23–28) had small sample sizes and it was not clear how they were otherwise biased, and the remaining 2 (21, 22) did not show significant other bias.

### 3.4 Outcome of intervention

**Figure 4:**
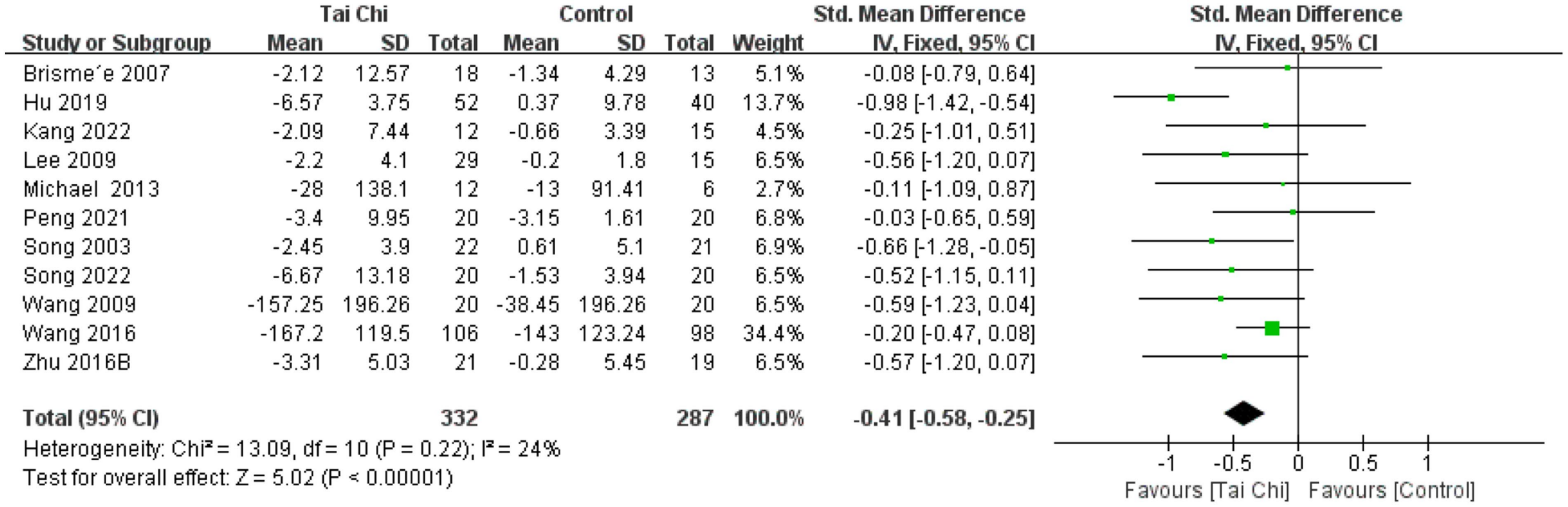
Forest plot of the effect of Tai Chi on WOMAC pain in patients with KOA.

**Figure 5:**
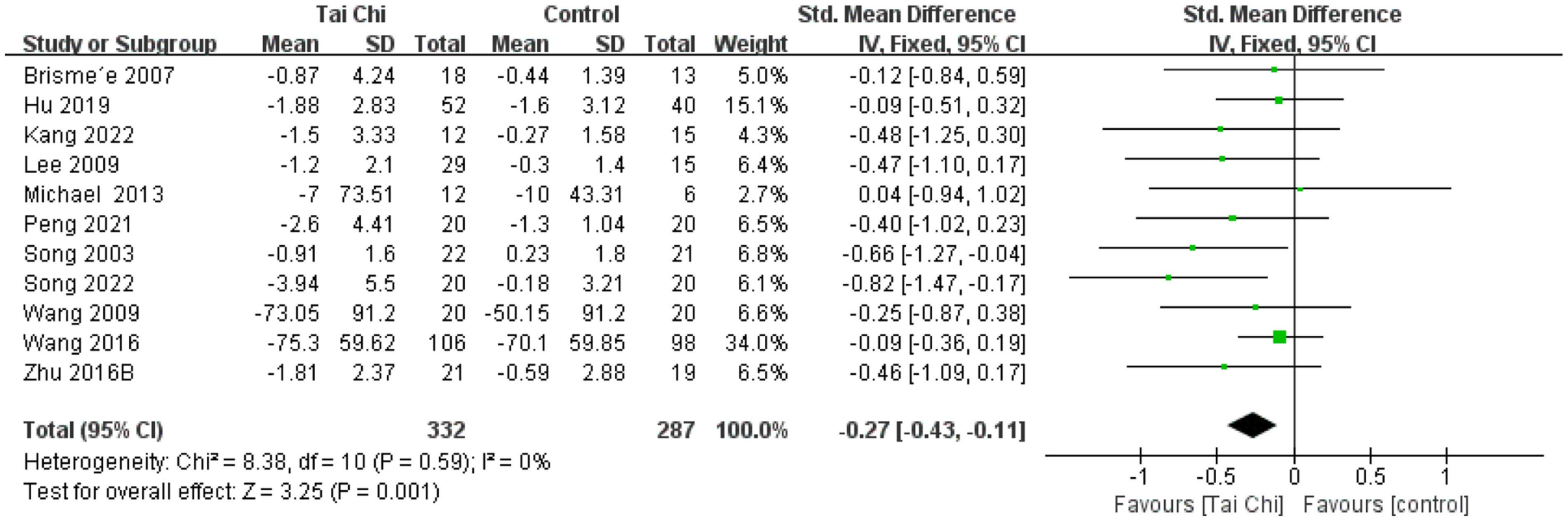
Forest plot of the effect of Tai Chi on WOMAC stiffness in patients with KOA.

**Figure 6:**
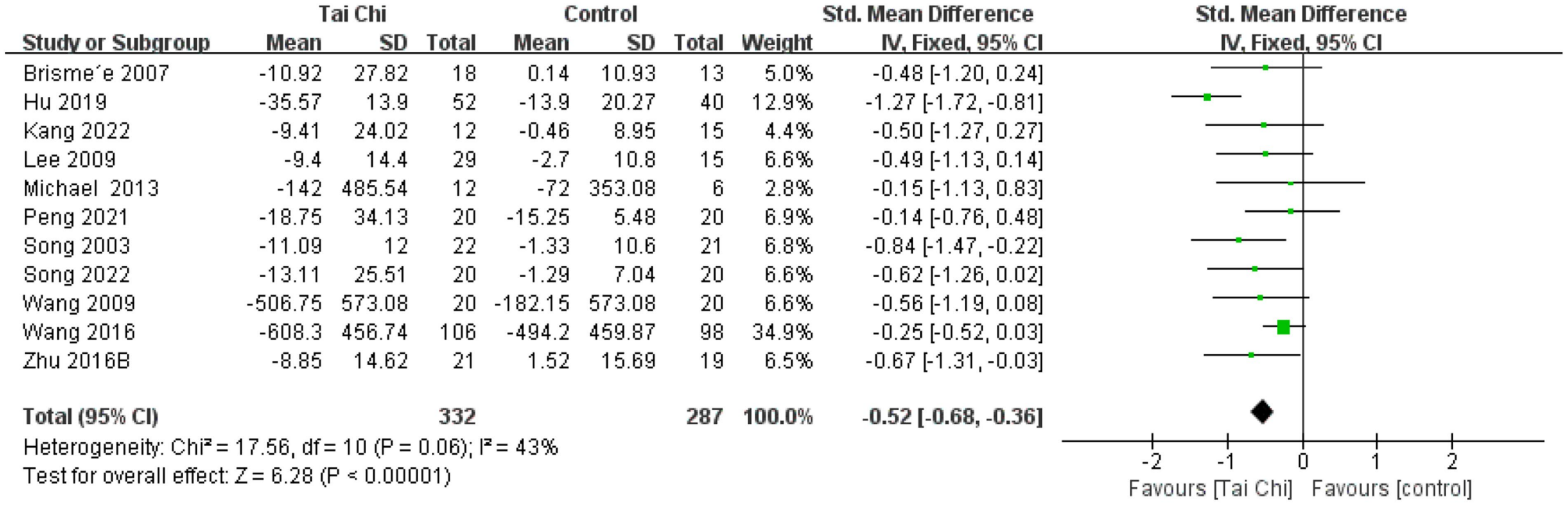
Forest plot of the effect of Tai Chi on WOMAC physical function in patients with KOA.

**Figure 7:**
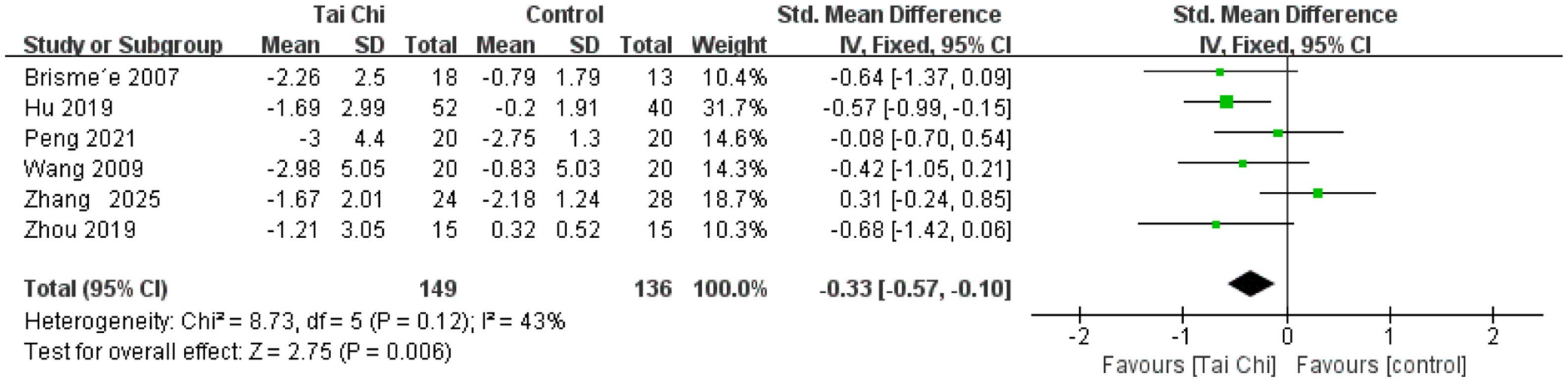
Forest plot of the effect of Tai Chi on VAS pain in patients with KOA.

**Figure 8:**
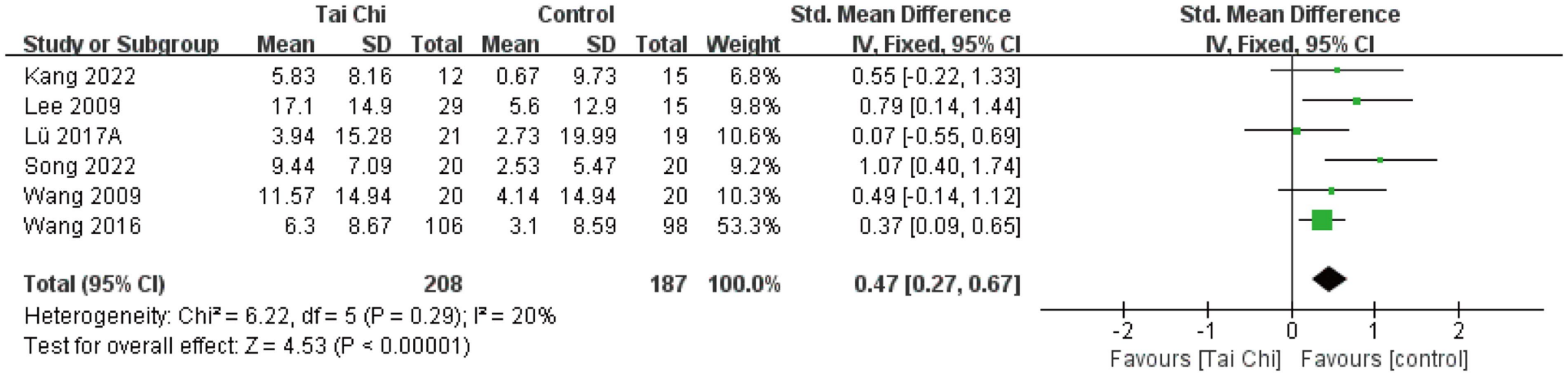
Forest plot of the effect of Tai Chi on SF-36 PCS in patients with KOA.

**Figure 9:**
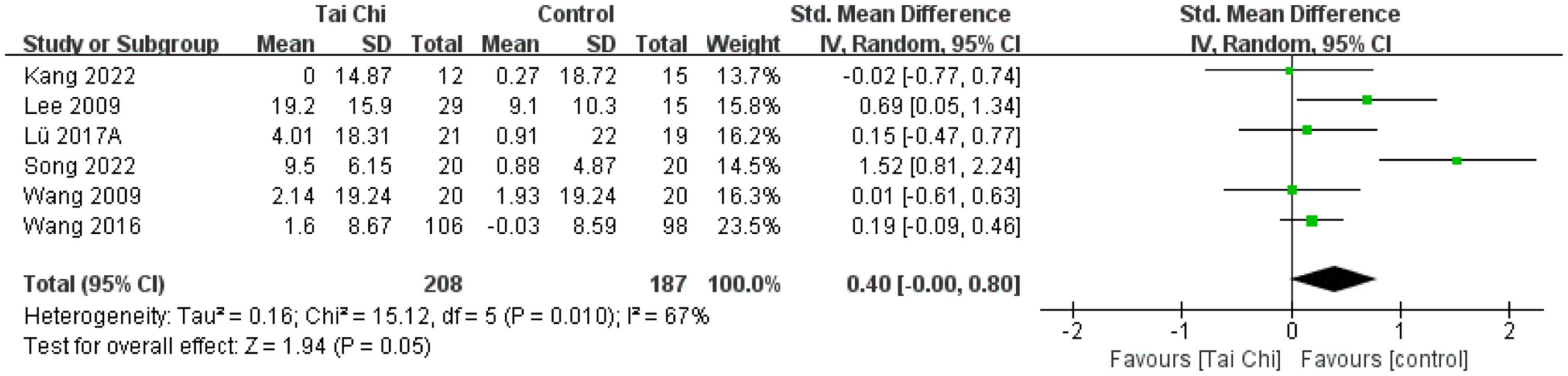
Forest plot of the effect of Tai Chi on SF-36 MCS in patients with KOA.

The 11 included studies used the WOMAC to measure pain, stiffness, and physical function, 5 studies used the VAS to measure pain, and 6 studies used the SF-36 to measure physical and mental health. Forest plots were drawn based on baseline and post-intervention outcomes (Figures 4–9). As different measurement tools were used, we calculated SMD and the 95% CI to standardize outcome data sizes.

#### 3.4.1 WOMAC pain

The 11 studies were analyzed for WOMAC pain, with a total of 619 participants. Overall results showed (Figure 4) that Tai Chi was significantly better than controls in improving pain (SMD = −0.41, 95% CI [−0.58, −0.25], *p* < 0.01). Meta-analysis showed low heterogeneity between studies (*I*^2^ = 24%, *p* = 0.22). Therefore, we combined the studies using a fixed-effects model.

#### 3.4.2 WOMAC stiffness

There were 11 studies analyzed for WOMAC stiffness with a total of 619 participants. Results showed (Figure 5) that Tai Chi was significantly better than the control group in improving stiffness (SMD = −0.27, 95% CI [−0.43, −0.11], *p* < 0.01). The overall results showed no heterogeneity between studies (*I*^2^ = 0%, *p* = 0.59). Therefore, we combined the studies using a fixed-effects model.

#### 3.4.3 WOMAC physical function

Eleven studies were analyzed for WOMAC physical function, with a total of 619 participants. The results showed (Figure 6) that Tai Chi was significantly better than the control group in improving physical function (SMD = −0.52, 95% CI [−0.68, −0.36], *p* < 0.01). Meta-analysis showed moderate heterogeneity between studies (*I*^2^ = 43%, *p* = 0.06). Therefore, we combined the studies using a fixed-effects model.

#### 3.4.4 VAS pain

Six studies were analyzed for VAS pain, with a total of 285 participants. The results showed (Figure 7) that Tai Chi was significantly better than the control group in improving pain (SMD = −0.33, 95%CI [−0.57, −0.10], *p* < 0.01). Overall results showed no heterogeneity between studies (*I*^2^ = 43%, *p* = 0.12). Therefore, we combined the studies using a fixed-effects model.

#### 3.4.5 SF-36 PCS

Physical health analyses were conducted on 6 studies with a total of 395 participants. Overall results (Figure 8) showed that Tai Chi was significantly better than the control group in improving physical health (SMD = 0.47, 95%CI [0.27, 0.67], *p* < 0.01). Meta-analysis showed low heterogeneity between studies (*I*^2^ = 24%, *p* = 0.29). Therefore, we combined the studies using a fixed-effects model.

#### 3.4.6 SF-36 MCS

Mental health analyses were conducted on 6 studies with a total of 395 participants. Overall results showed (Figure 9) that Tai Chi was significantly better than the control group in improving mental health (SMD = 0.40, 95%CI [−0.00, 0.80], *p* = 0.05). Meta-analysis showed significant heterogeneity between studies (*I*^2^ = 67%, *p* = 0.01). Therefore, we combined the studies using a random-effects model.

### 3.5 Subgroup analyses and GRADE evidence quality assessment

#### 3.5.1 Subgroup analyses

In order to explore in depth the optimal course and frequency of Tai Chi training protocols in terms of their effects on pain, stiffness, physical function, physical health, and mental health in patients with KOA, we categorized them into three protocols [short-term (≤16 weeks)/twice-weekly; short-term (≤16 weeks)/three-times-weekly; and long-term (>16 weeks)/three-times-weekly] based on the 13 included studies and performed subgroup analyses. Preliminary results indicate (Table 2; Supplementary Figure S1) that for improving pain and physical function, the long-term (>16 weeks)/three-times-weekly Tai Chi training protocol may be more suitable (SMD = −0.74, 95%CI [−1.06, −0.41], *p* < 0.01; SMD = −0.96, 95%CI [−1.30, −0.63], *p* < 0.01); for stiffness improvement, short-term (≤16 weeks)/three-times-weekly Tai Chi training was superior (SMD = −0.52; 95%CI [−0.84, −0.19], *p* < 0.01); for improving physical health, a short-term (≤16 weeks)/twice-weekly Tai Chi training regimen is recommended (SMD = 0.44, 95%CI [0.21, 0.68], *p* < 0.01).

**Table 2:** Subgroup analysis of the effect of Tai Chi in patients with KOA.

Index	Variable	Group	Sample size	Homogeneity test			Effect size and 95% CI	Test for overall effect	
				*C* ^2^	*p*	*I* ^2^		*Z*	*p*
WOMAC	Pain	ST,2×/wk	306	2.12	*p* = 0.55	0%	−0.29 [−0.52, −0.06]	2.51	*p* = 0.01
		ST,3×/wk	154	2.83	*p* = 0.42	0%	−0.34 [−0.66, −0.02]	2.07	*p* = 0.04
		LT, 3×/wk	159	3.02	*p* = 0.22	34%	−0.74 [−1.06, −0.41]	4.45	*p* < 0.00001
	Stiffness	ST,2×/wk	306	1.74	*p* = 0.63	0%	−0.16 [−0.38, 0.07]	1.35	*p* = 0.18
		ST,3×/wk	154	2.33	*p* = 0.51	0%	−0.52 [−0.84, −0.19]	3.14	*p* = 0.002
		LT, 3×/wk	159	1.29	*p* = 0.52	0%	−0.25 [−0.56, 0.07]	1.54	*p* = 0.12
	Function	ST,2×/wk	306	1.22	*p* = 0.75	0%	−0.32 [−0.54, −0.09]	2.71	*p* = 0.007
		ST,3×/wk	154	2.58	*p* = 0.46	0%	−0.52 [−0.85, −0.20]	3.15	*p* = 0.002
		LT, 3×/wk	159	3.90	*p* = 0.14	49%	−0.96 [−1.30, −0.63]	5.66	*p* < 0.00001
VAS pain		ST,2×/wk	122	5.33	*p* = 0.07	62%	−0.23 [−0.83, 0.37]	0.75	*p* = 0.45
		ST,3×/wk	71	1.33	*p* = 0.25	25%	−0.32 [−0.87, 0.23]	1.15	*p* = 0.25
SF-36	PCS	ST,2×/wk	288	1.41	*p* = 0.49	0%	0.44 [0.21, 0.68]	3.67	*p* = 0.0002
		LT, 3×/wk	67	0.91	*p* = 0.34	0%	0.26 [−0.23, 0.74]	1.04	*p* = 0.30
	MCS	ST,2×/wk	288	2.58	*p* = 0.27	23%	0.23 [−0.00, 0.46]	1.93	*p* = 0.05
		LT, 3×/wk	67	0.11	*p* = 0.74	0%	0.08 [−0.40, 0.57]	0.34	*p* = 0.73
Yang style	Pain	ST,2×/wk	262	1.33	*p* = 0.51	0%	−0.25 [−0.50, −0.01]	2.02	*p* = 0.04
		ST,3×/wk	71	0.83	*p* = 0.36	0%	−0.32 [−0.80, 0.15]	1.34	*p* = 0.18
	Stiffness	ST,2×/wk	262	0.30	*p* = 0.86	0%	−0.10 [−0.35, 0.14]	0.83	*p* = 0.41
		ST,3×/wk	71	1.99	*p* = 0.16	50%	−0.51 [−0.98, −0.03]	2.06	*p* = 0.04
	Function	ST,2×/wk	262	0.84	*p* = 0.66	0%	−0.29 [−0.53, −0.04]	2.30	*p* = 0.02
		ST,3×/wk	71	0.08	*p* = 0.78	0%	−0.56 [−1.04, −0.08]	2.29	*p* = 0.02

ST,2×/wk, short-term, twice a week; ST,3×/wk, short-term, three times a week; LT, 3×/wk, long-term, three times a week.

Among the 13 included studies, Yang-style Tai Chi was chosen for 6 studies. We noted that different courses and frequencies of Yang-style Tai Chi training protocols had different improvement effects on various symptoms in patients with KOA. Subgroup analyses showed (Table 2; Supplementary Figure S1) that a short-term (≤16 weeks)/twice-weekly Tai Chi training protocol was optimal in terms of pain improvement (SMD = −0.25, 95%CI [−0.50, −0.01], *p* = 0.04), and a short-term (≤16 weeks)/three-times-weekly Tai Chi training protocol was optimal in terms of stiffness and physical function improvement (SMD = −0.51; 95%CI [−0.98, −0.03], *p* = 0.04; SMD = −0.56; 95%CI [−1.04, −0.08], *p* = 0.02).

#### 3.5.2 GRADE evidence quality assessment

The GRADE software was used to assess the quality of evidence for the subgroup analyses of the three Tai Chi training protocols. Preliminary results (Table 3) demonstrated that among the 21 subgroup analysis outcomes, 7 were rated as low quality and 14 as moderate quality. Notably, the evidence for the Tai Chi training protocols we recommend was graded as moderate quality.

**Table 3:** GRADE results of subgroup analysis of the course and frequency of Tai Chi treatment for KOA.

Outcomes	Illustrative comparative risks* (95% CI)		Relative effect (95% CI)	No of participants (studies)	Quality of the evidence (GRADE)	Comments
	Assumed risk	Corresponding risk				
	Control	Subgroup analysis				
WOMAC Pain		The mean womac pain in the intervention groups was 0.41 standard deviations lower (0.58–0.25 lower)		619 (11 studies)	⊕⊕⊕⊝ moderate^1^	SMD −0.41 (−0.58 to −0.25)
WOMAC Pain—short-term, twice a week		The mean womac pain—short-term, twice a week in the intervention groups was 0.29 standard deviations lower (0.52–0.06 lower)		306 (4 studies)	⊕⊕⊕⊝ moderate^1^	SMD −0.29 (−0.52 to −0.06)
WOMAC Pain—short-term, three times a week		The mean womac pain—short-term, three times a week in the intervention groups was 0.34 standard deviations lower (0.66–0.02 lower)		154 (4 studies)	⊕⊕⊕⊝ moderate^1^	SMD −0.34 (−0.66 to −0.02)
WOMAC Pain—long-term, three times a week		The mean womac pain—long-term, three times a week in the intervention groups was 0.74 standard deviations lower (1.06–0.41 lower)		159 (3 studies)	⊕⊕⊕⊝ moderate^1^	SMD −0.74 (−1.06 to −0.41)
WOMAC stiffness		The mean womac stiffness in the intervention groups was 0.27 standard deviations lower (0.43–0.11 lower)		619 (11 studies)	⊕⊕⊕⊝ moderate^1^	SMD −0.27 (−0.43 to −0.11)
WOMAC stiffness—short-term, twice a week		The mean womac stiffness—short-term, twice a week in the intervention groups was 0.16 standard deviations lower (0.38 lower to 0.07 higher)		306 (4 studies)	⊕⊕⊝⊝ low^1,2^	SMD −0.16 (−0.38 to 0.07)
WOMAC stiffness—short-term, three times a week		The mean womac stiffness—short-term, three times a week in the intervention groups was 0.52 standard deviations lower (0.84–0.19 lower)		154 (4 studies)	⊕⊕⊕⊝ moderate^1^	SMD −0.52 (−0.84 to −0.19)
WOMAC stiffness—long-term, three times a week		The mean womac stiffness—long-term, three times a week in the intervention groups was 0.25 standard deviations lower (0.56 lower to 0.07 higher)		159 (3 studies)	⊕⊕⊝⊝ low^1,2^	SMD −0.25 (−0.56 to 0.07)
WOMAC function		The mean womac function in the intervention groups was 0.52 standard deviations lower (0.68–0.36 lower)		619 (11 studies)	⊕⊕⊕⊝ moderate^1^	SMD −0.52 (−0.68 to −0.36)
WOMAC function—short-term, twice a week		The mean womac function—short-term, twice a week in the intervention groups was 0.32 standard deviations lower (0.54–0.09 lower)		306 (4 studies)	⊕⊕⊕⊝ moderate^1^	SMD −0.32 (−0.54 to −0.09)
WOMAC function—short-term, three times a week		The mean womac function—short-term, three times a week in the intervention groups was 0.52 standard deviations lower (0.85–0.2 lower)		154 (4 studies)	⊕⊕⊕⊝ moderate^1^	SMD −0.52 (−0.85 to −0.2)
WOMAC function—long-term, three times a week		The mean womac function—long-term, three times a week in the intervention groups was 0.96 standard deviations lower (1.3–0.63 lower)		159 (3 studies)	⊕⊕⊕⊝ moderate^1^	SMD −0.96 (−1.3 to −0.63)
VAS pain		The mean vas pain in the intervention groups was 0.26 standard deviations lower (0.63–0.12 lower)		193 (5 studies)	⊕⊕⊕⊝ moderate^1^	SMD −0.26 (−0.63 to 0.12)
VAS pain—short-term, twice a week		The mean vas pain—short-term, twice a week in the intervention groups was 0.23 standard deviations lower (0.83–0.37 lower)		122 (3 studies)	⊕⊕⊕⊝ moderate^1^	SMD −0.23 (−0.83 to 0.37)
VAS pain- short-term, three times a week		The mean vas pain- short-term, three times a week in the intervention groups was 0.32 standard deviations lower (0.87 lower to 0.23 higher)		71 (2 studies)	⊕⊕⊝⊝ low^1,2^	SMD −0.32 (−0.87 to 0.23)
SF-36 PCS		The mean sf-36 pcs in the intervention groups was 0.41 standard deviations higher (0.19–0.62 higher)		355 (5 studies)	⊕⊕⊕⊝ moderate^1^	SMD 0.41 (0.19–0.62)
SF-36 PCS—short-term, twice a week		The mean sf-36 pcs—short-term, twice a week in the intervention groups was 0.44 standard deviations higher (0.21–0.68 higher)		288 (3 studies)	⊕⊕⊕⊝ moderate^1^	SMD 0.44 (0.21–0.68)
SF-36 PCS—long-term, three times a week		The mean sf-36 pcs—long-term, three times a week in the intervention groups was 0.26 standard deviations higher (0.23 lower to 0.74 higher)		67 (2 studies)	⊕⊕⊝⊝ low^1,2^	SMD 0.26 (−0.23 to 0.74)
SF-36 MCS		The mean sf-36 mcs in the intervention groups was 0.2 standard deviations higher (0.01 lower to 0.41 higher)		355 (5 studies)	⊕⊕⊝⊝ low^1,2^	SMD 0.2 (−0.01 to 0.41)
SF-36 MCS—short-term, twice a week		The mean sf-36 mcs—short-term, twice a week in the intervention groups was 0.23 standard deviations higher (0–0.46 higher)		288 (3 studies)	⊕⊕⊕⊝ moderate^1^	SMD 0.23 (0–0.46)
SF-36 MCS—long-term, three times a week		The mean sf-36 mcs—long-term, three times a week in the intervention groups was 0.08 standard deviations higher (0.4 lower to 0.57 higher)		67 (2 studies)	⊕⊕⊝⊝ low^1,2^	SMD 0.08 (−0.4 to 0.57)
Yang-style Tai Chi— WOMAC pain		The mean yang-style tai chi— womac pain in the intervention groups was 0.27 standard deviations lower (0.48–0.05 lower)		333 (5 studies)	⊕⊕⊕⊝ moderate^1^	SMD −0.27 (−0.48 to −0.05)
Yang-style Tai Chi—WOMAC pain—short-term, twice a week		The mean yang-style tai chi—womac pain—short-term, twice a week in the intervention groups was 0.25 standard deviations lower (0.5–0.01 lower)		262 (3 studies)	⊕⊕⊕⊝ moderate^1^	SMD −0.25 (−0.5 to −0.01)
Yang-style Tai Chi—WOMAC pain—short-term, three times a week		The mean yang-style tai chi—womac pain—short-term, three times a week in the intervention groups was 0.32 standard deviations lower (0.8 lower to 0.15 higher)		71 (2 studies)	⊕⊕⊝⊝ low^1,2^	SMD −0.32 (−0.8 to 0.15)
Yang-style Tai Chi—WOMAC stiffness		The mean yang-style tai chi—womac stiffness in the intervention groups was 0.19 standard deviations lower (0.4 lower to 0.03 higher)		333 (5 studies)	⊕⊕⊝⊝ low^1,2^	SMD −0.19 (−0.4 to 0.03)
Yang-style Tai Chi—WOMAC stiffness—short-term, twice a week		The mean yang-style tai chi—womac stiffness—short-term, twice a week in the intervention groups was 0.1 standard deviations lower (0.35 lower to 0.14 higher)		262 (3 studies)	⊕⊕⊝⊝ low^1,2^	SMD −0.1 (−0.35 to 0.14)
Yang-style Tai Chi—WOMAC stiffness—short-term, three times a week		The mean yang-style tai chi—womac stiffness—short-term, three times a week in the intervention groups was 0.51 standard deviations lower (0.98–0.03 lower)		71 (2 studies)	⊕⊕⊕⊝ moderate^1^	SMD −0.51 (−0.98 to −0.03)
Yang-style Tai Chi—WOMAC function		The mean yang-style tai chi—womac function in the intervention groups was 0.34 standard deviations lower (0.56–0.13 lower)		333 (5 studies)	⊕⊕⊕⊝ moderate^1^	SMD −0.34 (−0.56 to −0.13)
Yang-style Tai Chi—WOMAC function—short-term, twice a week		The mean yang-style tai chi—womac function—short-term, twice a week in the intervention groups was 0.29 standard deviations lower (0.53–0.04 lower)		262 (3 studies)	⊕⊕⊕⊝ moderate^1^	SMD −0.29 (−0.53 to −0.04)
Yang-style Tai Chi—WOMAC function—short-term, three times a week		The mean yang-style tai chi—womac function—short-term, three times a week in the intervention groups was 0.56 standard deviations lower (1.04–0.08 lower)		71 (2 studies)	⊕⊕⊕⊝ moderate^1^	SMD −0.56 (−1.04 to −0.08)

Subgroup analysis for optimal course and frequency.

Patient or population: patients with optimal course and frequency.

Intervention: Subgroup analysis.

*The basis for the assumed risk (e.g., the median control group risk across studies) is provided in footnotes. The corresponding risk (and its 95% confidence interval) is based on the assumed risk in the comparison group and the relative effect of the intervention (and its 95% CI).

CI: Confidence interval.

GRADE Working Group grades of evidence.

High quality: Further research is very unlikely to change our confidence in the estimate of effect.

Moderate quality: Further research is likely to have an important impact on our confidence in the estimate of effect and may change the estimate.

Low quality: Further research is very likely to have an important impact on our confidence in the estimate of effect and is likely to change the estimate.

Very low quality: We are very uncertain about the estimate.

^1^All studies were randomized, some did not mention that the allocation was hidden, and some were likely to be blinded during implementation.

^2^ The sample size is small or the confidence interval is wide.

### 3.6 Sensitivity analysis

We performed sensitivity analyses on the WOMAC, VAS, and SF-36 results of the 13 included studies to assess the robustness of the overall results. The results showed that the data points of the studies fell within the effect sizes of the original confidence intervals, indicating the stability of the analyzed results. Supplementary Figure S2, provides detailed results of the sensitivity analysis.

### 3.7 Evaluation of publication bias

**Figure 10:**
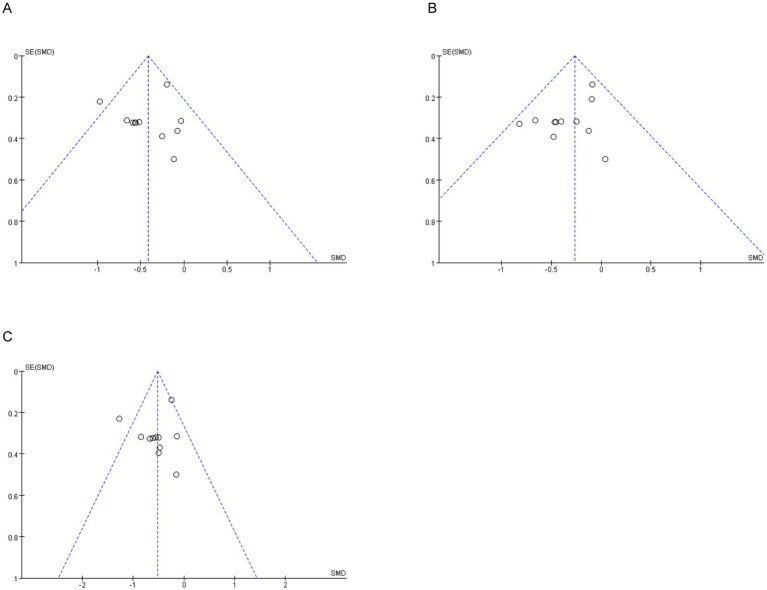
Funnel plot.

According to the Cochrane Handbook for Systematic Reviews, at least 10 included studies are needed to test for funnel plot asymmetry. Therefore, only data on WOMAC pain, WOMAC stiffness, and WOMAC physical function were available for analysis. As shown (Figure 10), the funnel plots for WOMAC pain, WOMAC stiffness, and WOMAC physical function were almost symmetrically distributed. Subsequently, Egger’s tests were performed for these three variables, and the Egger’s test results for WOMAC pain, WOMAC stiffness, and WOMAC physical function were *p* = 0.75, *p* = 0.07, *p* = 0.57, respectively (Supplementary Table S6). Overall results indicated that there was no publication bias in the intervention effects of Tai Chi on pain, stiffness and physical function.

## 4 Discussion

### 4.1 Overall findings

The potential mechanism of Tai Chi for KOA involves multi-system synergy. At the physiological level, Tai Chi promotes blood circulation around the knee joint through slow and fluid movements in conjunction with breathing and reduces the levels of local pro-inflammatory factors (e.g., Interleukin-6, and Tumor Necrosis Factor-α), thereby reducing inflammation-mediated pain and cartilage degeneration (29). In terms of neuro-immuno-modulation, Tai Chi can simultaneously modulate the resting-state functional connectivity of the descending opioidergic pathway and reward/motivation system and blood inflammation markers, which is important for the prevention of joint damage (30). In terms of psychological mechanisms, Tai Chi’s integration of meditation and dynamic relaxation also has positive effects on psychological states (31, 32). Although the available evidence supports the combined benefits of Tai Chi in improving KOA symptoms, quality of life, and psychological state, more research is needed to validate the specific mechanisms.

The present study confirmed by meta-analysis that Tai Chi significantly improves pain, stiffness, physical function and physical health in patients with KOA. Tai Chi demonstrated a moderate effect size of improvement in pain, physical function and physical health in patients with KOA. Although the effect size of Tai Chi on stiffness improvement was small, the statistical significance still demonstrated the positive effect of Tai Chi on improving stiffness. Subgroup analysis further indicates the optimal “dose” of Tai Chi for treating different symptoms of KOA. Preliminary findings suggest that for improving pain and physical function, a long-term (>16 weeks)/three-times-weekly training protocol is recommended; for alleviating stiffness, a short-term (≤16 weeks)/three-times-weekly protocol is advised; and for enhancing physical health, a short-term (≤16 weeks)/twice-weekly protocol may be more appropriate.

The results of this review are consistent with those of Gao and Tan (33, 34), who reported the effectiveness of Tai Chi in relieving pain and stiffness and improving physical function and physical health. The findings of this study are also consistent with the ACR guideline recommendation of “Tai Chi as a safe complementary therapy for KOA,” but with a breakthrough in the optimization of treatment course and frequency. Previous meta-analyses have focused on the overall efficacy of Tai Chi versus a control group, but have not explored the impact of intervention parameters. For example, Li’s study only analyzed subgroups by dividing sessions into 8 weeks versus 12 weeks and did not incorporate frequency (35). Qiu’s study demonstrated that both long-term (>12 weeks) and short-term (≤12 weeks) therapeutic exercise interventions yielded significant improvements in WOMAC-assessed pain, stiffness, physical function, and SF-36 physical health scores when compared to the control group, also without incorporating frequency (36).

Sensitivity analysis results indicate the stability of our findings. However, we observed moderate heterogeneity in MCS and VAS pain for the short-term/twice-weekly regimen (*I*^2^ = 67%, *I*^2^ = 62%). After excluding studies one by one, we found that the heterogeneity for MCS decreased to 0% when Song et al. (10) was removed (Supplementary Figure S3). We speculate the primary reasons are as follows: First, the study samples spanned China, the United States, and South Korea, regions exhibiting significant differences in mental health assessment standards. Second, substantial variations existed in the Tai Chi training course and frequency; only Song’s study employed a 12-week/three-times-weekly protocol, while other studies utilized differing protocols. Finally, the depth of mind–body integration during Tai Chi practice may vary depending on instructor proficiency and implementation settings. The heterogeneity for short-term/twice-weekly VAS pain decreased to 0% after excluding Zhang et al. (28) (Supplementary Figure S4). This was most probably because the control group received balance and postural control training, reducing the effect size difference between the intervention and control groups. Although the heterogeneity in both results was moderate, sensitivity analysis indicated the stability of this study, making the findings of this meta-analysis relatively reliable.

Although this study provides evidence supporting the efficacy of Tai Chi for KOA, we acknowledge that clinical heterogeneity exists among the various control groups—including health education, physical therapy, attention control, herbal intervention, balance and postural control training, and no intervention—which is an important limitation. The differences between these control interventions may influence effect assessments in two key aspects: First, potential overestimation of effect size: Tai Chi’s efficacy may appear amplified when compared against controls like “no intervention.” Using physical therapy—itself a recommended treatment for KOA—as a control group could diminish the observed effect size. Second, challenges in generalizability: The diversity of control measures reflects the wide variation in KOA management practices in real-world clinical settings, complicating direct comparisons across studies. Despite these limitations, our findings align with the review by Gao and Tan, both reporting Tai Chi’s efficacy in alleviating pain and stiffness while improving physical function and physiological health. Future RCTs should standardize control groups to minimize bias.

Among the various Tai Chi styles, Yang-style Tai Chi is the most commonly used intervention (37). Of the 13 RCTs included in this study, 6 studies selected Yang-style Tai Chi. A Yang-style Tai Chi training program is optimal for short-term (≤16 weeks)/twice-weekly Tai Chi training programs in improving pain, and short-term (≤16 weeks)/three-times-weekly Tai Chi training programs in improving stiffness and physical function. Except for the course and frequency of the intervention in improving stiffness, which was consistent with that of general Tai Chi, the rest of the protocols were inconsistent (general Tai Chi was considered optimal in improving pain and physical function over a long-term (16 > weeks)/three-times-weekly), which may be related to factors such as the small number of included studies and the small sample size (For example, subgroup analysis of the short-term Yang-style Tai Chi training protocol with thrice-weekly sessions included only two studies totaling 71 participants). This may affect the reliability of our recommendations for optimal treatment protocols. Regarding Tai Chi styles, detailed analysis was conducted solely on Yang-style Tai Chi due to insufficient data representativeness for other styles such as Chen-style and Sun-style. This study was lacking in direct comparisons between different styles, constituting a significant research limitation. Future research should validate whether specific styles of Tai Chi are more effective for KOA by comparing different styles.

Although this study provides evidence-based recommendations for Tai Chi interventions in KOA management, three key factors warrant discussion. First, regarding implementation details, 11 of the 13 included RCTs featured 60 min per session, with 10 studies employing professional Tai Chi instructors. Practice formats varied between group and individual settings, as well as indoor and outdoor locations. Notably, among the 13 studies, 6 explicitly used the standardized 24-form Yang-style Tai Chi, 1 used Chen-style, 1 used Sun-style, while the remaining studies lacked descriptions of the Tai Chi style. This heterogeneity emphasizes the need for future trials to report the following: (1) the precise Tai Chi form/sequence, (2) session duration/intensity metrics, and (3) instructor expertise level. These details are crucial for clinical replication. Second, reporting inconsistencies existed regarding confounding factors like combined therapies and lifestyle modifications. Only one study explicitly stated no changes to routine physical activity or medication during the intervention period, while the remaining studies made no mention of any confounding factors. These gaps underscore the need to minimize potential confounding factors in future study designs to reduce their impact on results. Third, only four studies in this meta-analysis included follow-up assessments, all of which involved 12-week Tai Chi course. Wang et al. (22) reported sustained benefits up to 52 weeks, whereas Wang et al. (25) and Song et al. (10) indicated near-statistical significance at 24 weeks. We recommend that future RCTs include ≥6 months of follow-up to assess durability of effects and conduct cost-effectiveness analyses comparing sustained versus intermittent Tai Chi interventions. These enhancements will strengthen clinical translation, enabling precise specification of Tai Chi parameters (style, dose, supervision). This should account for real-world comorbidities and lifestyle factors, particularly among older adults with multiple conditions requiring polypharmacy. An optimized Tai Chi protocol may become a sustainable cornerstone in non-pharmacological KOA management.

It is noteworthy that when designing a Tai Chi training protocol, clinicians should be flexible in choosing the frequency and course of training based on the patient’s specific situation and preferences. For patients who have limited time or are physically weak, twice-weekly training may be more appropriate. Although the efficacy is not as good as three-times-weekly training program, it can still bring some relief. For those patients who are able to spend more time and energy, three-times-weekly training may bring more comprehensive efficacy. In conclusion, Tai Chi training should be implemented in conjunction with individualized guidance and supervision to ensure the quality and safety of the training. In this way, Tai Chi can not only serve as a complementary therapy for KOA patients, but also become part of their daily lives, helping them to improve their quality of life. However, only one study in this meta-analysis reported adverse events. Due to the limited number of adverse event reports, it is not possible to draw definitive conclusions about the safety of Tai Chi, highlighting the need for improved safety reporting in future trials.

### 4.2 Limitations and future research directions

There are several limitations to this study. First, 13 RCTs were included in this review and meta-analysis, but the sample sizes of these studies were small, which could easily lead to unstable results. Second, most of the RCTs utilized a single-blind design, which may have had an impact on the objectivity of the results. Third, the longest intervention period of the included studies was 36 weeks, and there was a lack of follow-up data to assess the long-term effects and recurrence rates of Tai Chi. Fourth, as different schools and styles of Tai Chi practice may have different effects on the results, and this study only analyzed Yang-style Tai Chi in detail without comparing different schools. Finally, although the optimal course and frequency of Tai Chi practice were reported in this study, the patients’ individual circumstances, including the strengths and weaknesses of their physical fitness, the severity of their illnesses, and the duration of their illnesses, were not adequately taken into account. To comprehensively evaluate the efficacy of Tai Chi therapy for KOA, determine optimal treatment course and frequency, future studies should expand their scale, incorporate long-term follow-up assessments, standardize reporting of adverse events, adopt double-blind designs to reduce bias, and unify control group interventions to minimize heterogeneity. Additionally, research should focus on refining intervention details and standardizing Tai Chi practice types to facilitate comparison of results across studies. Finally, it is recommended that future studies combine biomarker and imaging assessments to more accurately measure the specific effects of Tai Chi training on patients with KOA.

## 5 Conclusion

This study suggests that Tai Chi is an effective non-pharmacological treatment for improving KOA symptoms, and adopts a “symptom-oriented” Tai Chi intervention strategy. Preliminary results from this meta-analysis suggest that long-term high-frequency protocols may be more suitable for patients experiencing pain and functional limitations, while short-term high-frequency protocols may be preferable for those with stiffness. For individuals requiring physical conditioning, short-term low-frequency protocols may be more appropriate. Although the current level of evidence for the recommended approach is moderate, this study has limitations including small sample size, limited blinding, lack of long-term follow-up, and insufficient reporting of adverse events. Future high-quality research is needed to strengthen the evidence base and promote the widespread application of Tai Chi in the management of KOA.

## Data Availability

The original contributions presented in the study are included in the article/Supplementary material, further inquiries can be directed to the corresponding author.
