# Supplementary material for: The optimal course and frequency of Tai Chi for knee osteoarthritis: a systematic review and meta-analysis of randomized controlled trials
Source: Front Public Health. 2025 Oct 28;13:1661674. doi: 10.3389/fpubh.2025.1661674 (PMC12602386; doi:10.3389/fpubh.2025.1661674)

**The optimal course and frequency of Tai Chi for Knee Osteoarthritis: A Systematic Review and Meta-Analysis of Randomized Controlled Trials**

Content

**S1 Table. PRISMA 3**

**S2 Table. Search strategy 7**

**S3 Table. Raw data and calculation of mean and SD for all RCTs 8**

**S4 Table. Excluded studies and reasons 18**

**S5 Table. Reasons for risk of bias 21**

**S6 Table. Egger’s test for each outcome 22**

**S1 Figure. Subgroup analysis of the effect of Tai Chi on WOMAC pain/WOMAC stiffness/WOMAC 26**

**S2 Figure. Sensitivity Analysis 28**

**S3 Figure. Heterogeneity after excluding Song (2022) 30**

**S4 Figure. Heterogeneity after excluding Zhang（2025） 30**

S1 Table. PRISMA

| **Section and Topic** | **Item #** | **Checklist item** | **Location where item is reported** |
| --- | --- | --- | --- |
| **TITLE** | | |  |
| Title | 1 | Identify the report as a systematic review. | Title |
| **ABSTRACT** | | |  |
| Abstract | 2 | See the PRISMA 2020 for Abstracts checklist. | Abstract |
| **INTRODUCTION** | | |  |
| Rationale | 3 | Describe the rationale for the review in the context of existing knowledge. | Introduction |
| Objectives | 4 | Provide an explicit statement of the objective(s) or question(s) the review addresses. | Introduction |
| **METHODS** | | |  |
| Eligibility criteria | 5 | Specify the inclusion and exclusion criteria for the review and how studies were grouped for the syntheses. | Inclusion and Exclusion Criteria,Statistical Analysis |
| Information sources | 6 | Specify all databases, registers, websites, organisations, reference lists and other sources searched or consulted to identify studies. Specify the date when each source was last searched or consulted. | Methods,Search Strategy |
| Search strategy | 7 | Present the full search strategies for all databases, registers and websites, including any filters and limits used. | Search Strategy |
| Selection process | 8 | Specify the methods used to decide whether a study met the inclusion criteria of the review, including how many reviewers screened each record and each report retrieved, whether they worked independently, and if applicable, details of automation tools used in the process. | Study Selection |
| Data collection process | 9 | Specify the methods used to collect data from reports, including how many reviewers collected data from each report, whether they worked independently, any processes for obtaining or confirming data from study investigators, and if applicable, details of automation tools used in the process. | Data Extraction |
| Data items | 10a | List and define all outcomes for which data were sought. Specify whether all results that were compatible with each outcome domain in each study were sought (e.g. for all measures, time points, analyses), and if not, the methods used to decide which results to collect. | Data Extraction |
|  | 10b | List and define all other variables for which data were sought (e.g. participant and intervention characteristics, funding sources). Describe any assumptions made about any missing or unclear information. | Data Extraction |
| Study risk of bias assessment | 11 | Specify the methods used to assess risk of bias in the included studies, including details of the tool(s) used, how many reviewers assessed each study and whether they worked independently, and if applicable, details of automation tools used in the process. | Risk of bias assessment and GRADE |
| Effect measures | 12 | Specify for each outcome the effect measure(s) (e.g. risk ratio, mean difference) used in the synthesis or presentation of results. | Statistical Analysis |
| Synthesis methods | 13a | Describe the processes used to decide which studies were eligible for each synthesis (e.g. tabulating the study intervention characteristics and comparing against the planned groups for each synthesis (item #5)). | Statistical Analysis |
|  | 13b | Describe any methods required to prepare the data for presentation or synthesis, such as handling of missing summary statistics, or data conversions. | Statistical Analysis |
|  | 13c | Describe any methods used to tabulate or visually display results of individual studies and syntheses. | Statistical Analysis |
|  | 13d | Describe any methods used to synthesize results and provide a rationale for the choice(s). If meta-analysis was performed, describe the model(s), method(s) to identify the presence and extent of statistical heterogeneity, and software package(s) used. | Statistical Analysis |
|  | 13e | Describe any methods used to explore possible causes of heterogeneity among study results (e.g. subgroup analysis, meta-regression). | Statistical Analysis |
|  | 13f | Describe any sensitivity analyses conducted to assess robustness of the synthesized results. | Statistical Analysis |
| Reporting bias assessment | 14 | Describe any methods used to assess risk of bias due to missing results in a synthesis (arising from reporting biases). | Assessment of Reporting Quality |
| Certainty assessment | 15 | Describe any methods used to assess certainty (or confidence) in the body of evidence for an outcome. | Statistical Analysis |
| **RESULTS** | | |  |
| Study selection | 16a | Describe the results of the search and selection process, from the number of records identified in the search to the number of studies included in the review, ideally using a flow diagram. | Search Results |
|  | 16b | Cite studies that might appear to meet the inclusion criteria, but which were excluded, and explain why they were excluded. | Search Results |
| Study characteristics | 17 | Cite each included study and present its characteristics. | Study Characteristics |
| Risk of bias in studies | 18 | Present assessments of risk of bias for each included study. | Assessment of Risk of Bias |
| Results of individual studies | 19 | For all outcomes, present, for each study: (a) summary statistics for each group (where appropriate) and (b) an effect estimate and its precision (e.g. confidence/credible interval), ideally using structured tables or plots. | Outcome of Intervention |
| Results of syntheses | 20a | For each synthesis, briefly summarise the characteristics and risk of bias among contributing studies. | Study Characteristics，Assessment of Risk of Bias |
|  | 20b | Present results of all statistical syntheses conducted. If meta-analysis was done, present for each the summary estimate and its precision (e.g. confidence/credible interval) and measures of statistical heterogeneity. If comparing groups, describe the direction of the effect. | Outcome of Intervention |
|  | 20c | Present results of all investigations of possible causes of heterogeneity among study results. | Overall Findings |
|  | 20d | Present results of all sensitivity analyses conducted to assess the robustness of the synthesized results. | Sensitivity Analysis |
| Reporting biases | 21 | Present assessments of risk of bias due to missing results (arising from reporting biases) for each synthesis assessed. | Evaluation of Publication Bias |
| Certainty of evidence | 22 | Present assessments of certainty (or confidence) in the body of evidence for each outcome assessed. | GRADE evidence quality assessment |
| **DISCUSSION** | | |  |
| Discussion | 23a | Provide a general interpretation of the results in the context of other evidence. | Overall Findings |
|  | 23b | Discuss any limitations of the evidence included in the review. | Limitations and future research directions |
|  | 23c | Discuss any limitations of the review processes used. | Overall Findings，Limitations and future research directions |
|  | 23d | Discuss implications of the results for practice, policy, and future research. | Conclusions |
| **OTHER INFORMATION** | | |  |
| Registration and protocol | 24a | Provide registration information for the review, including register name and registration number, or state that the review was not registered. | PROSPERO registration information |
|  | 24b | Indicate where the review protocol can be accessed, or state that a protocol was not prepared. | None |
|  | 24c | Describe and explain any amendments to information provided at registration or in the protocol. | None |
| Support | 25 | Describe sources of financial or non-financial support for the review, and the role of the funders or sponsors in the review. | Funding |
| Competing interests | 26 | Declare any competing interests of review authors. | Competing interests |
| Availability of data, code and other materials | 27 | Report which of the following are publicly available and where they can be found: template data collection forms; data extracted from included studies; data used for all analyses; analytic code; any other materials used in the review. | Availability of data and materials |

S2 Table. Search strategy

| **Database** | **Search strategy** | |
| --- | --- | --- |
| **PubMed** | **#1** | "osteoarthritis, knee" [MeSH Terms] OR ("osteoarthritis knee" [Title/Abstract] OR (("Knee" [MeSH Terms] OR "Knee"[All Fields] OR "knee joint" [MeSH Terms] OR ("Knee" [All Fields] AND "joint" [All Fields]) OR "knee joint" [All Fields]) AND "Osteoarthritides" [Title/Abstract]) OR "knee pain" [Title/Abstract] OR "knee joint osteoarthritis" [Title/Abstract] OR "knee arthritis" [Title/Abstract] OR "knee osteoarthritis" [Title/Abstract] OR "knee osteoarthrosis" [Title/Abstract] OR "osteoarthritis of the knee" [Title/Abstract] OR "osteoarthritis of knee" [Title/Abstract] OR "knee osteo arthritis" [Title/Abstract] OR "KOA" [Title/Abstract] OR "Gonarthrosis" [Title/Abstract])  Translations  Knee: "knee" [MeSH Terms] OR "knee" [All Fields] OR"knee joint" [MeSH Terms] OR ("knee" [All Fields] AND "joint" [All Fields]) OR "knee joint" [All Fields] |
|  | **#2** | "tai ji"[MeSH Terms] OR "tai ji"[Title/Abstract] OR "tai chi" [Title/Abstract] OR "chi tai" [Title/Abstract] OR "tai chi chuan" [Title/Abstract] OR "Taiji" [Title/Abstract] OR "Taijiquan" [Title/Abstract] OR "t ai chi" [Title/Abstract] OR "tai ji quan" [Title/Abstract] OR "ji quan tai" [Title/Abstract] OR "quan tai ji"[Title/Abstract] |
|  | **#3** | "Randomized Controlled Trials as Topic"[MeSH Terms] OR "randomized controlled trial"[Title/Abstract] OR "clinical trials randomized"[Title/Abstract] OR "trials randomized clinical" [Title/Abstract] OR "controlled clinical trials" [Title/Abstract] OR "controlled clinical trial"[Title/Abstract] OR "Randomized" [Title/Abstract] OR "Randomized" [Title/Abstract] OR "trial" [Title/Abstract] OR "placebo"[Title/Abstract] |
|  | **#1AND #2 AND #3** | |
| **Embase** | **#1** | 'knee osteoarthritis'/exp OR 'arthrosis, knee':ab,ti OR gonarthrosis:ab,ti OR 'knee arthrosis':ab,ti OR 'knee joint arthrosis':ab,ti OR 'knee joint osteoarthritis':ab,ti OR 'knee osteo-arthritis':ab,ti OR 'knee osteo-arthrosis':ab,ti OR 'knee osteoarthrosis':ab,ti OR 'osteoarthritis, knee':ab,ti OR 'osteoarthrosis, knee':ab,ti OR 'knee osteoarthritis':ab,ti OR koa:ab,ti |
|  | **#2** | 'tai chi'/exp OR 'tai chi chuan':ab,ti OR 'taiji quan':ab,ti OR 'tai chi':ab,ti OR taiji:ab,ti OR taichi:ab,ti OR taijiquan:ab,ti OR 'taiji':ab,ti |
|  | **#3** | 'randomized controlled trial (topic)'/exp OR 'pragmatic clinical trials as topic':ab,ti OR 'randomized controlled trials as topic':ab,ti OR ('randomized controlled trial':ab,ti AND topic:ab,ti) OR 'randomized controlled trials':ab,ti OR 'clinical trials, randomized':ab,ti OR 'trials,randomized clinical':ab,ti OR 'controlled clinical trials':ab,ti OR 'controlled clinical trial':ab,ti |
|  | **#1AND #2 AND #3** | |
| **Cochrane Library** | **#1** | MeSH descriptor: [Osteoarthritis, Knee] explode all trees OR (Osteoarthritis, Knee):ti,ab,kw OR (Osteoarthritis of the Knee):ti,ab,kw OR (Osteoarthritis of Knee):ti,ab,kw OR (Knee Osteoarthritis):ti,ab,kw OR (Knee Osteoarthritides):ti,ab,kw OR Knee joint Osteoarthritis):ti,ab,kw OR (Knee arthritis):ti,ab,kw OR (knee osteo-arthritis):ti,ab,kw OR (Gonarthrosis):ti,ab,kw OR (KOA):ti,ab,kw |
|  | **#2** | MeSH descriptor: [Tai Ji] explode all trees OR (Tai Ji):ti,ab,kw OR  (Taiji):ti,ab,kw OR (T'ai Chi):ti,ab,kw OR (Chi, Tai):ti,ab,kw OR (Tai Chi  Chuan):ti,ab,kw OR (Ji Quan, Tai):ti,ab,kw OR (Taijiquan):ti,ab,kw OR  (Tai Ji Quan):ti,ab,kw OR (Tai-ji):ti,ab,kw OR (Tai Chi):ti,ab,kw OR (Quan, Tai Ji):ti,ab,kw |
|  | **#3** | MeSH descriptor: [Randomized Controlled Trial] explode all trees OR  (Randomized Controlled Trial):ti,ab,kw OR (Controlled Clinical Trials,  Randomized):ti,ab,kw OR (Trials, Randomized Clinical):ti,ab,kw OR (Clinical Trials, Randomized):ti,ab,kw OR (randomized):ti,ab,kw |
|  | **#1AND #2 AND #3** | |
| **Web of science** | **#1** | ((((((((((((TS=(Osteoarthritis, Knee)) OR TS=(Osteoarthritis, Knee)) OR TS=(Knee Osteoarthritides)) OR TS=(Knee pain)) OR TS=(Knee joint Osteoarthritis)) OR TS=(Knee arthritis)) OR TS=(Knee Osteoarthritis)) OR TS=(knee osteoarthrosis)) OR TS=(Osteoarthritis of the Knee)) OR TS=(Osteoarthritis of Knee)) OR TS=(knee osteo-arthritis)) OR TS=(KOA)) OR TS=(Gonarthrosis) |
|  | **#2** | ((((((((((TS=(Tai Ji)) OR TS=(Tai-ji)) OR TS=(Tai Chi)) OR TS=(Chi, Tai)) OR TS=(Tai Chi Chuan)) OR TS=(Taiji)) OR TS=(Taijiquan)) OR TS=(T'ai Chi)) OR TS=(Tai Ji Quan)) OR TS=(Ji Quan, Tai)) OR TS=(Quan, Tai Ji) |
|  | **#3** | ((((((TS=(Randomized Controlled Trials)) OR TS=(randomized controlled trial)) OR TS=(Clinical Trials, Randomized)) OR TS=(Trials, Randomized Clinical)) OR TS= (Controlled Clinical Trials)) OR TS=(controlled clinical trial)) OR TS=(randomized) |
|  | **#1AND #2 AND #3** | |
| **Scopus** | **#1** | ( TITLE-ABS-KEY ( osteoarthritis, AND knee ) OR TITLE-ABS-KEY ( k nee AND osteoarthritides ) OR TITLE-ABS-KEY ( knee AND pain ) OR TITLE-ABS-KEY ( knee AND joint AND osteoarthritis ) OR TITLE-ABS -KEY ( knee AND arthritis ) OR TITLE-ABS-KEY ( knee AND osteoarth ritis ) OR TITLE-ABS-KEY ( knee AND osteoarthrosis ) OR TITLE-ABS -KEY ( osteoarthritis AND of AND the AND knee ) OR TITLE-ABS-KE Y ( osteoarthritis AND of AND knee ) OR TITLE-ABS-KEY ( knee AND osteo-arthritis ) OR TITLE-ABS-KEY ( gonarthrosis ) OR TITLE-ABS-K EY ( knee AND joint AND arthrosis ) ) |
|  | **#2** | ( TITLE-ABS-KEY ( tai AND ji ) OR TITLE-ABS-KEY ( tai-ji ) OR TIT LE-ABS-KEY ( tai AND chi ) OR TITLE-ABS-KEY ( chi, AND tai ) OR TITLE-ABS-KEY ( tai AND chi AND chuan ) OR TITLE-ABS-KEY ( tai ji ) OR TITLE-ABS-KEY ( taijiquan ) OR TITLE-ABS-KEY ( t'ai AND c hi ) OR TITLE-ABS-KEY ( tai AND ji quan ) OR TITLE-ABS-KEY ( ji AND quan, AND tai ) OR TITLE-ABS-KEY ( quan, tai AND ji ) ) |
|  | **#3** | ( TITLE-ABS-KEY ( randomized AND controlled AND trials ) OR TITLE-ABS-KEY ( randomized AND controlled AND trial ) OR TITLE-ABS-KEY ( clinical AND trials, AND randomized ) OR TITLE-ABS-KEY ( trials, AND randomized AND clinical ) OR TITLE-ABS-KEY ( controlled AND clinical AND trials ) OR TITLE-ABS-KEY ( controlled AND clinical AND trial ) OR TITLE-ABS-KEY ( randomized ) ) |
|  | **#1AND #2 AND #3** | |
| **EBSCO** | **#1** | SU osteoarthritis knee OR AB ( osteoarthritis knee OR Osteoarthritis, Knee OR Knee Osteoarthritides OR Knee pain OR Knee joint Osteoarthritis OR Knee arthritis OR Knee Osteoarthritis OR knee osteoarthrosis OR Osteoarthritis of the Knee OR Osteoarthritis of Knee OR knee osteo-arthritis OR Gonarthrosis ) |
|  | **#2** | SU Tai Ji OR AB ( Tai-ji OR Tai Chi OR Chi, Tai OR Tai Chi Chuan OR Taiji OR Taijiquan OR T'ai Chi OR Tai Ji Quan OR Ji Quan, Tai OR Quan, Tai Ji ) |
|  | **#3** | SU Randomized Controlled Trials OR AB ( RCT OR rct OR randomized controlled trial OR Clinical Trials, Randomized OR Trials, Randomized Clinical OR Controlled Clinical Trials OR controlled clinical trial OR randomized OR Randomized OR trial ) |
|  | **#1AND #2 AND #3** | |
| **Chinese database** | **#1** | 骨关节炎（Osteoarthritis） OR 肌骨骼疾病（musculoskeletal disorders）OR 关节疾病（joint disorders） |
|  | **#2** | 膝（Knee） |
|  | **#3** | 太极拳（Taijiquan）OR太极（Taiji） |
|  | **#4** | 随机对照试验（Randomised controlled trial）OR临床研究（Clinical study）OR临床试验（Clinical trial）OR研究（Study） |
|  | **#1AND #2 AND #3AND #4** | |

S3 Table. Raw data and calculation of mean and SD for all RCTs

See Raw data and calculation of mean and SD for all RCTs—Excel

S4 Table. Excluded studies and reasons

**Records excluded based on title/abstract，with reasons(n=359)**

**-Retracted article(n=4)**


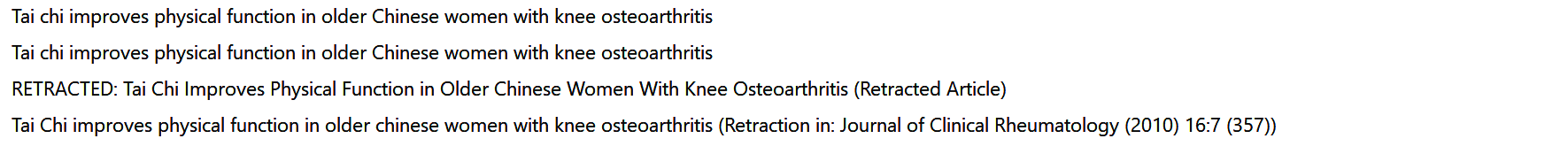


**-Not  RCT(n=261)**


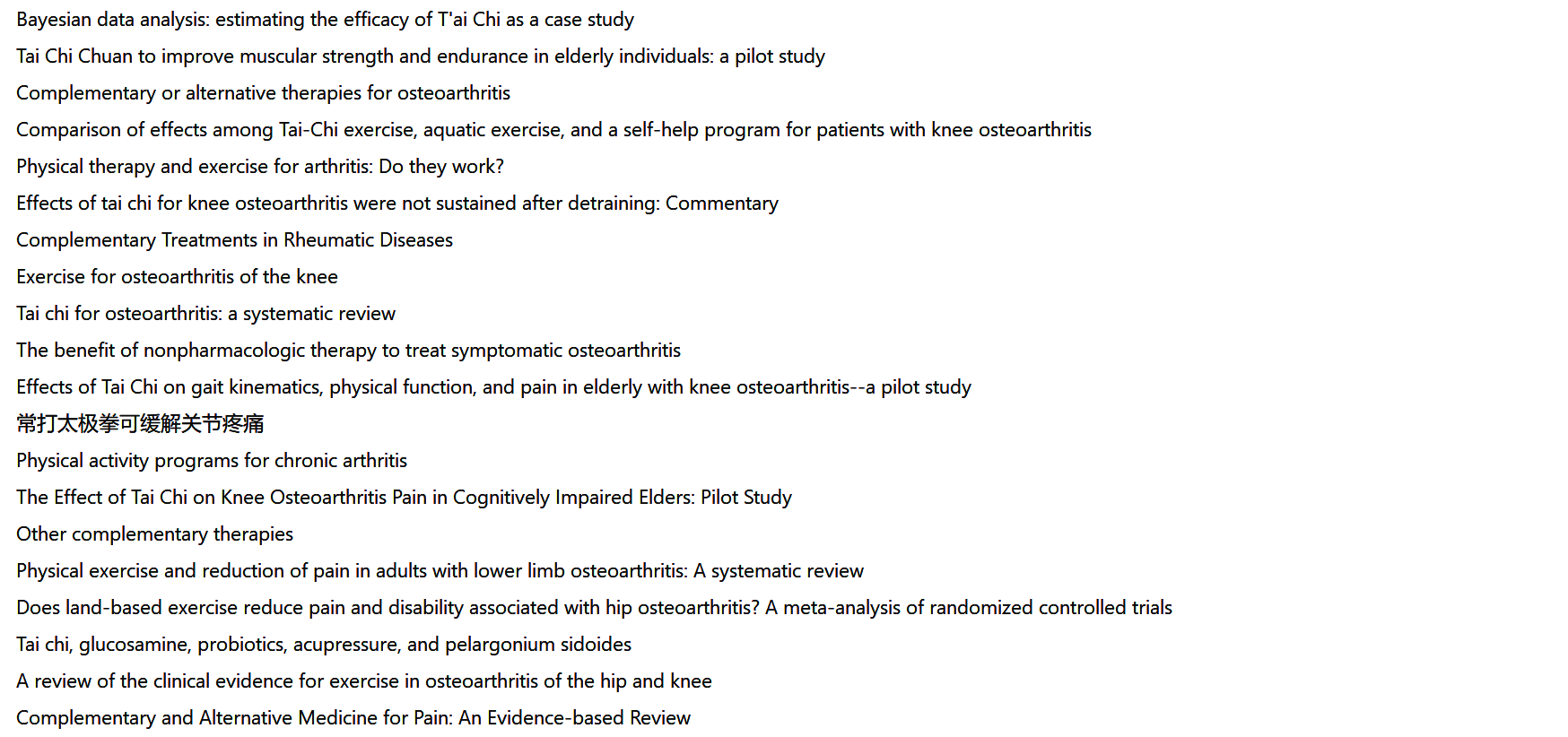

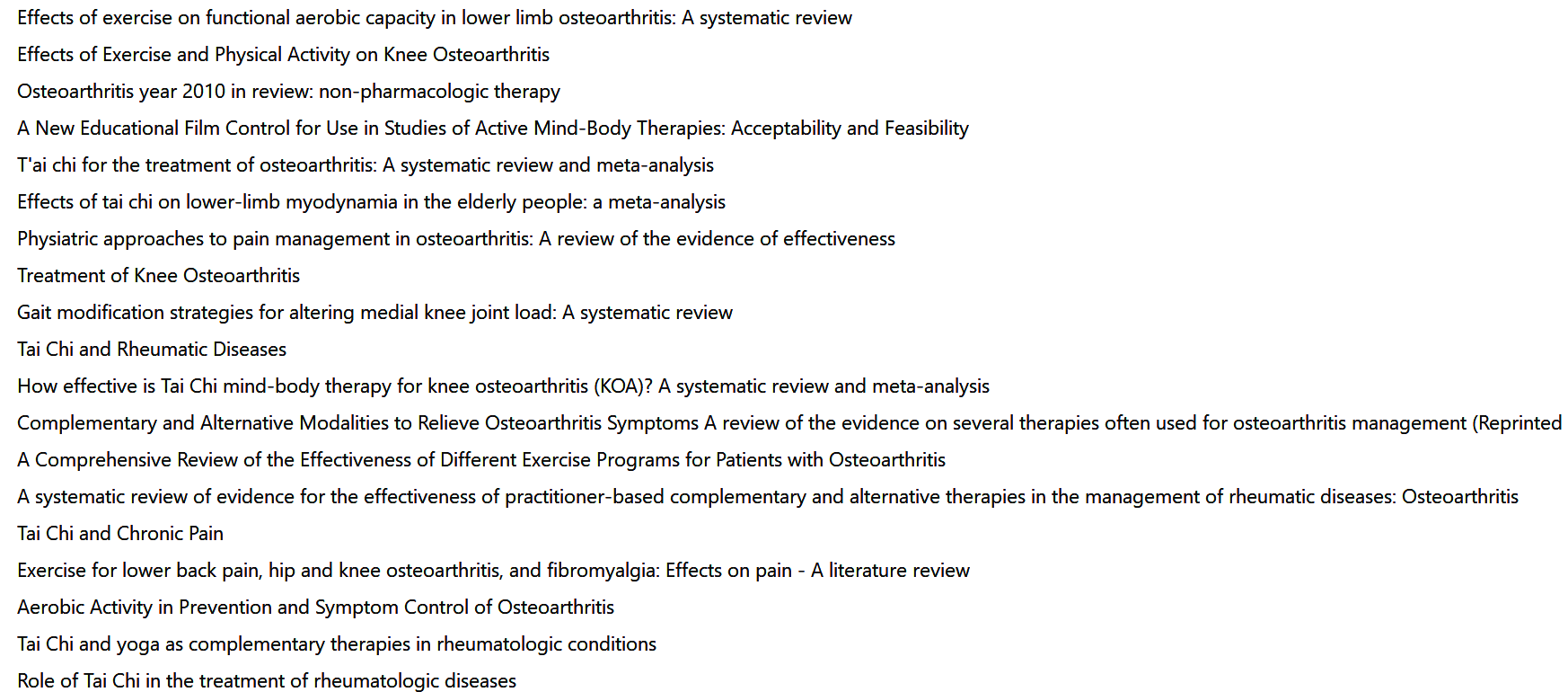

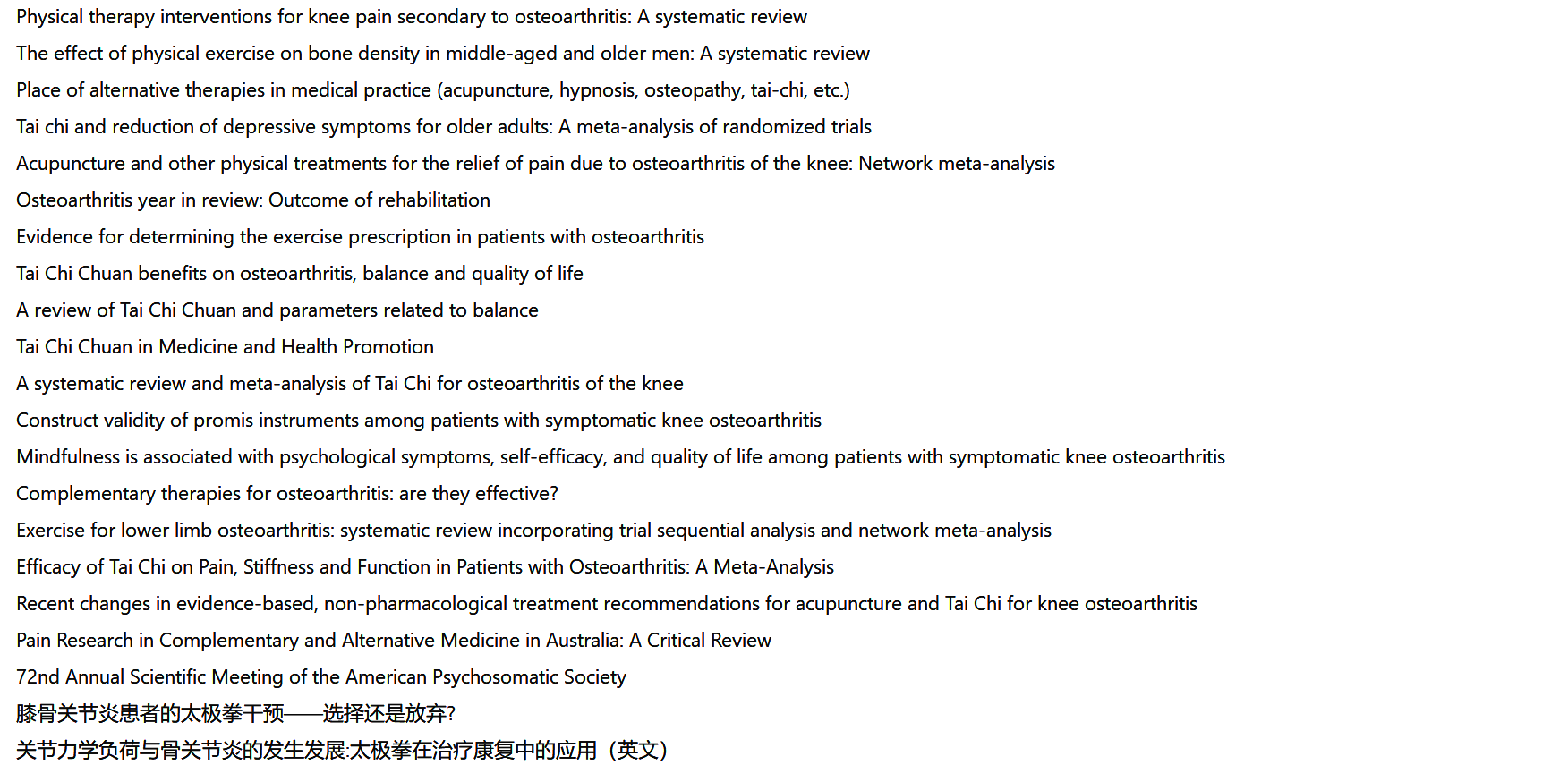

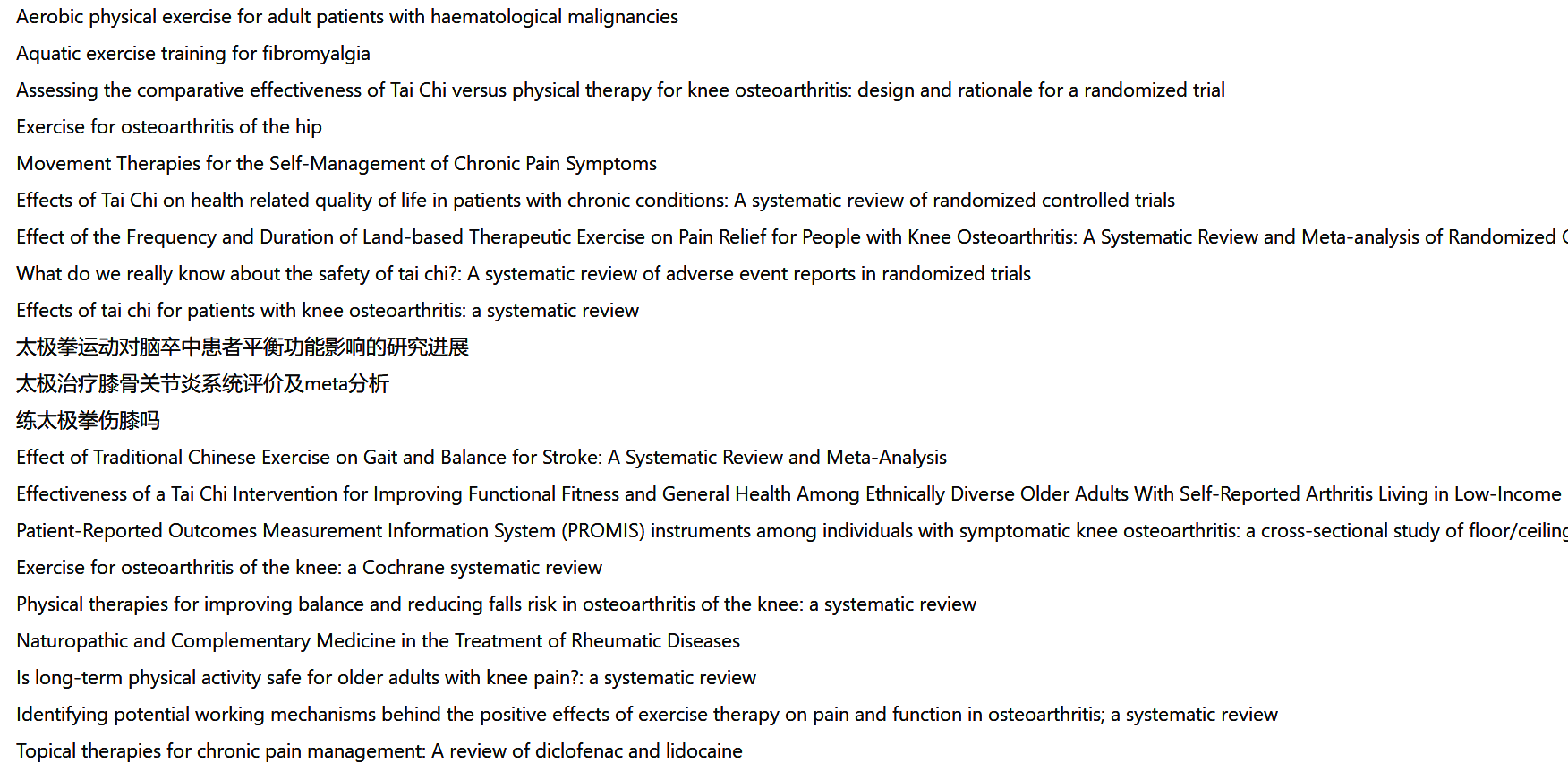

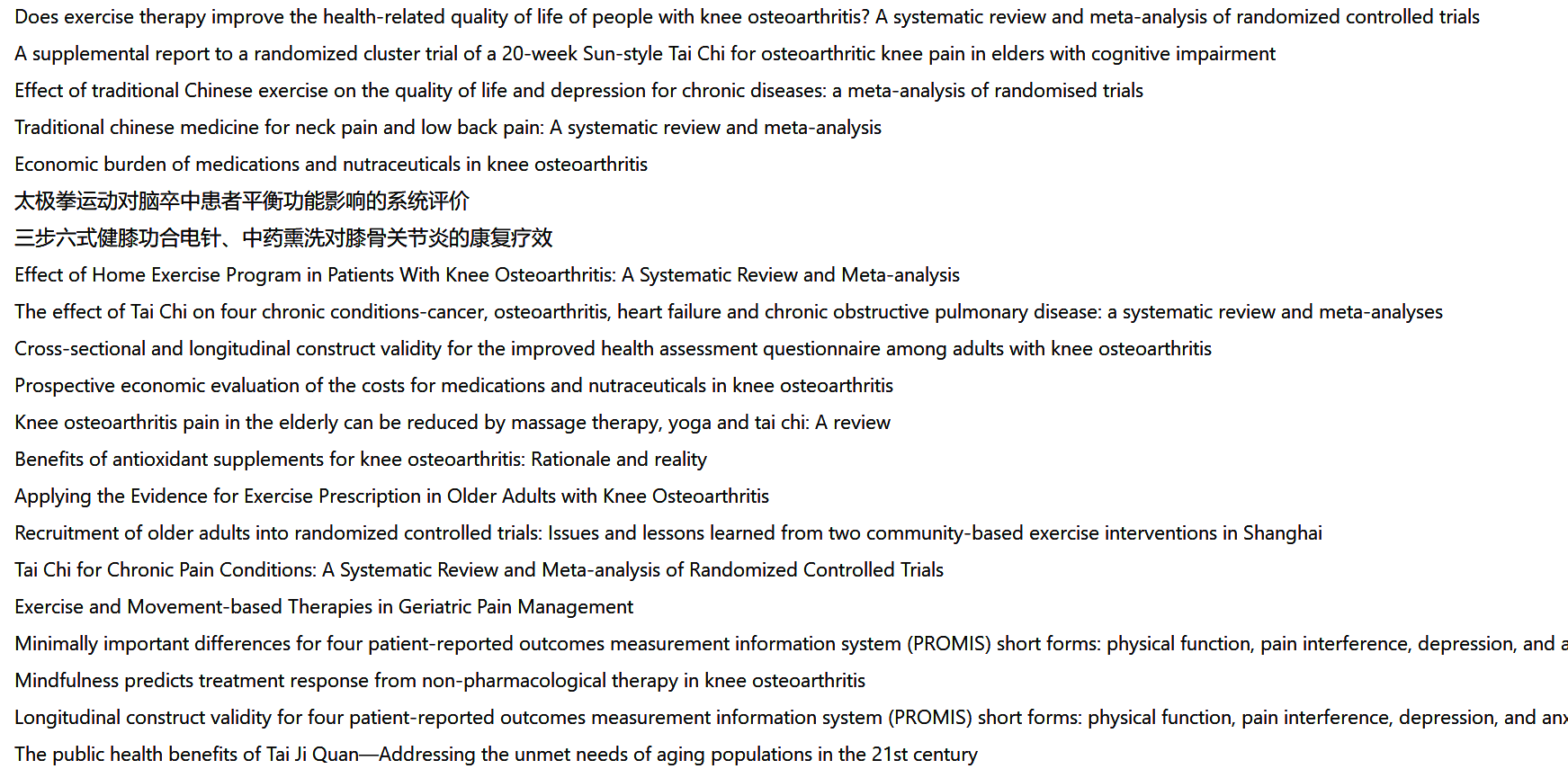

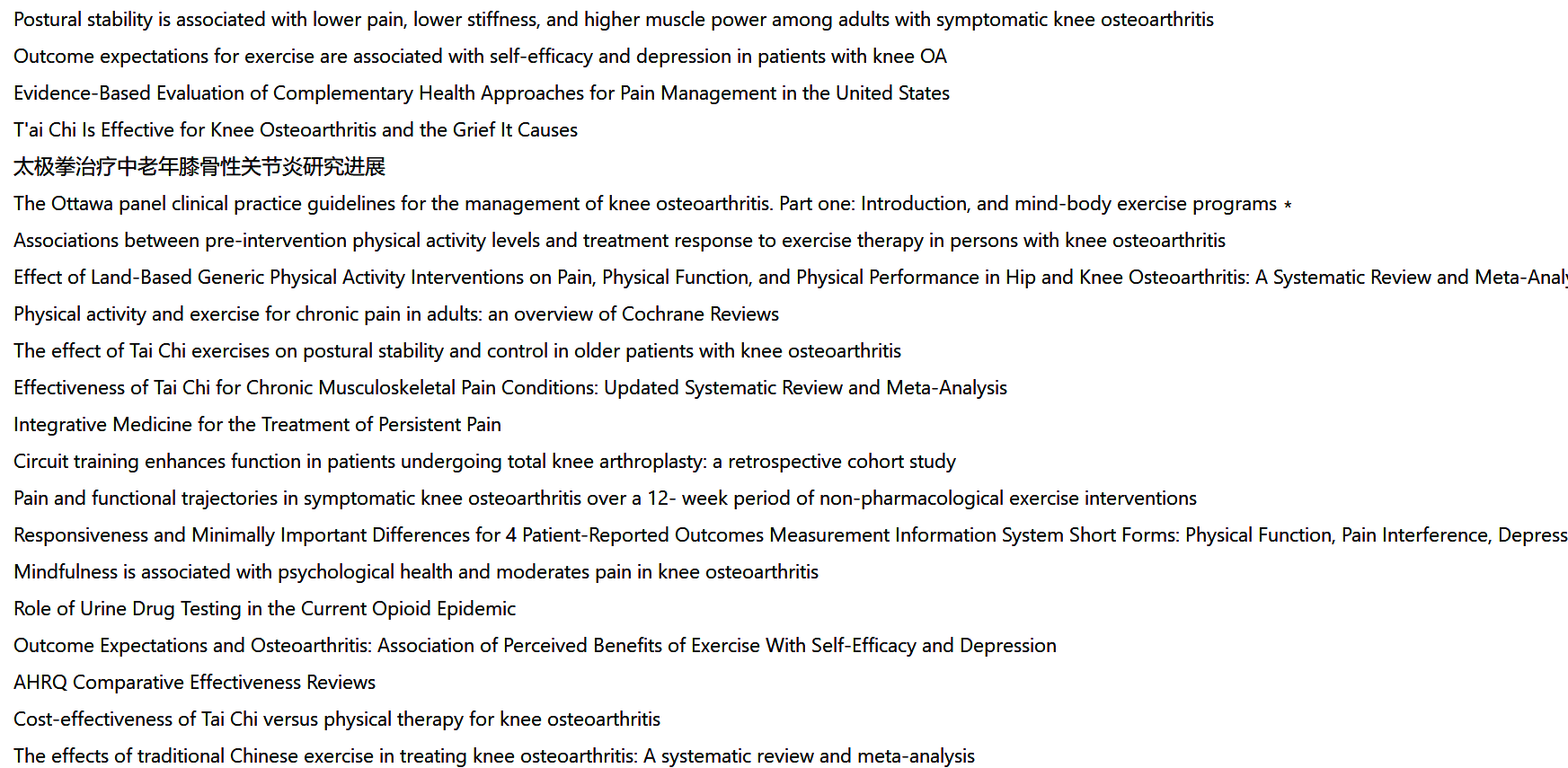

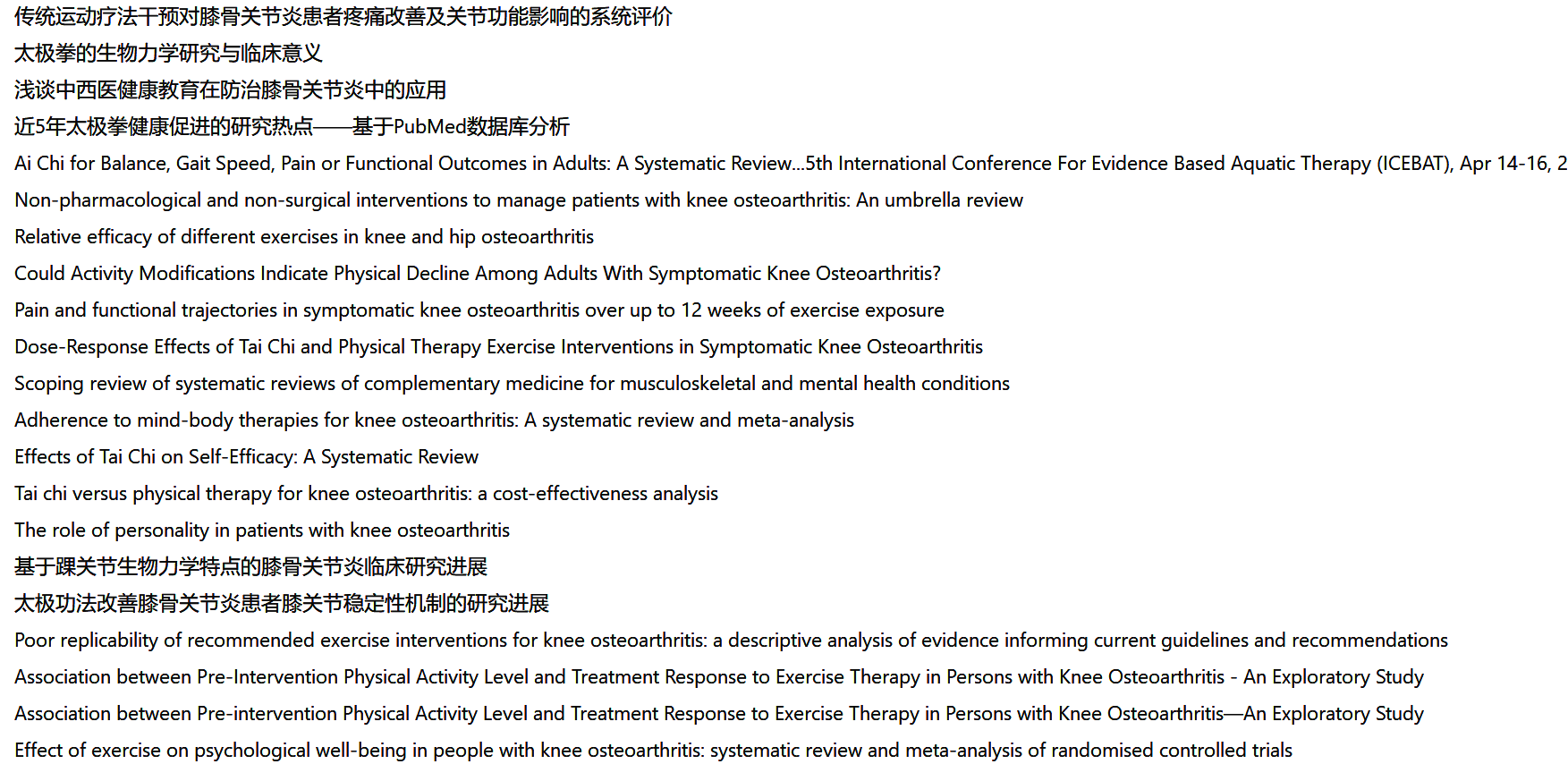

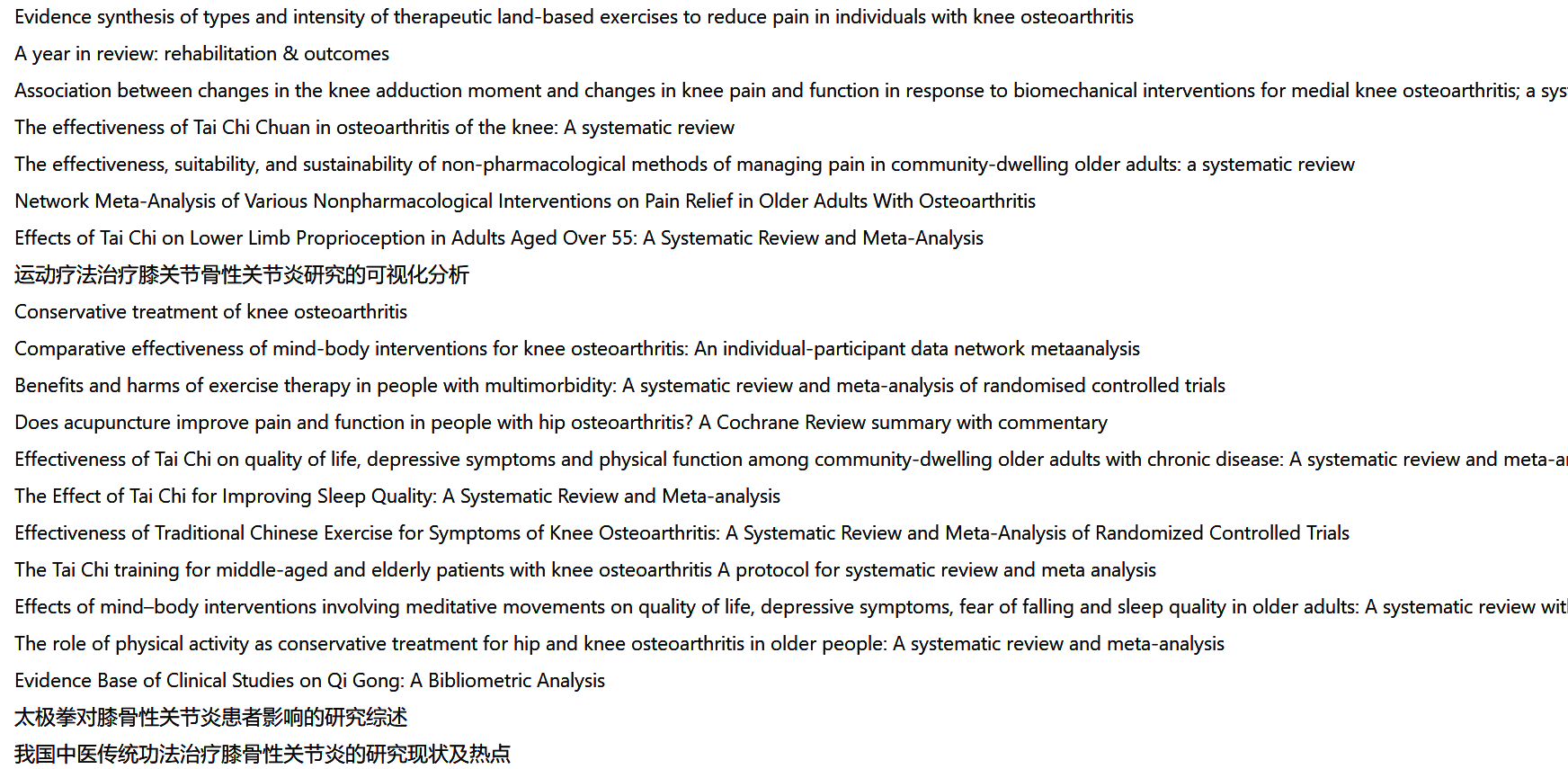

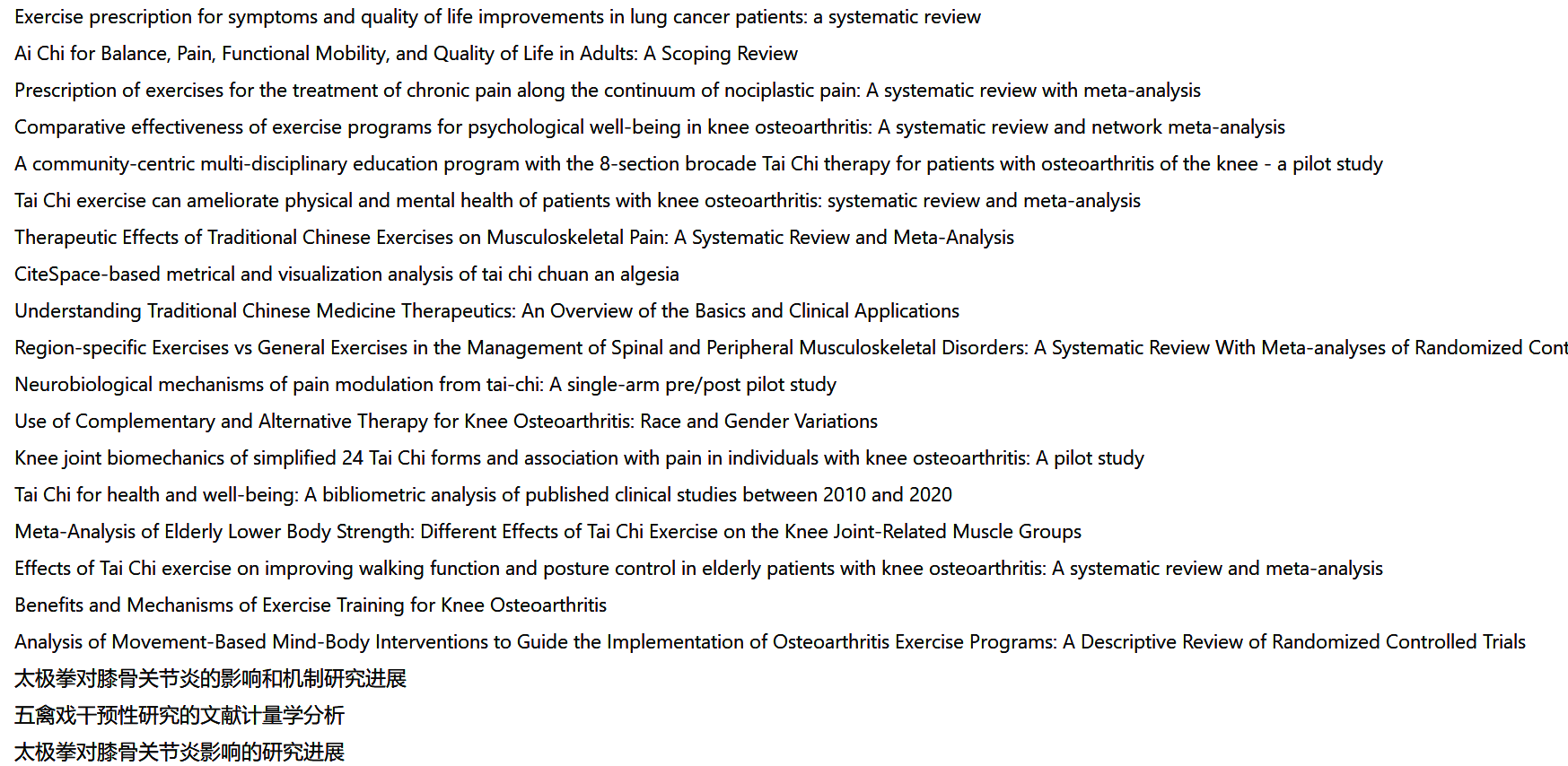

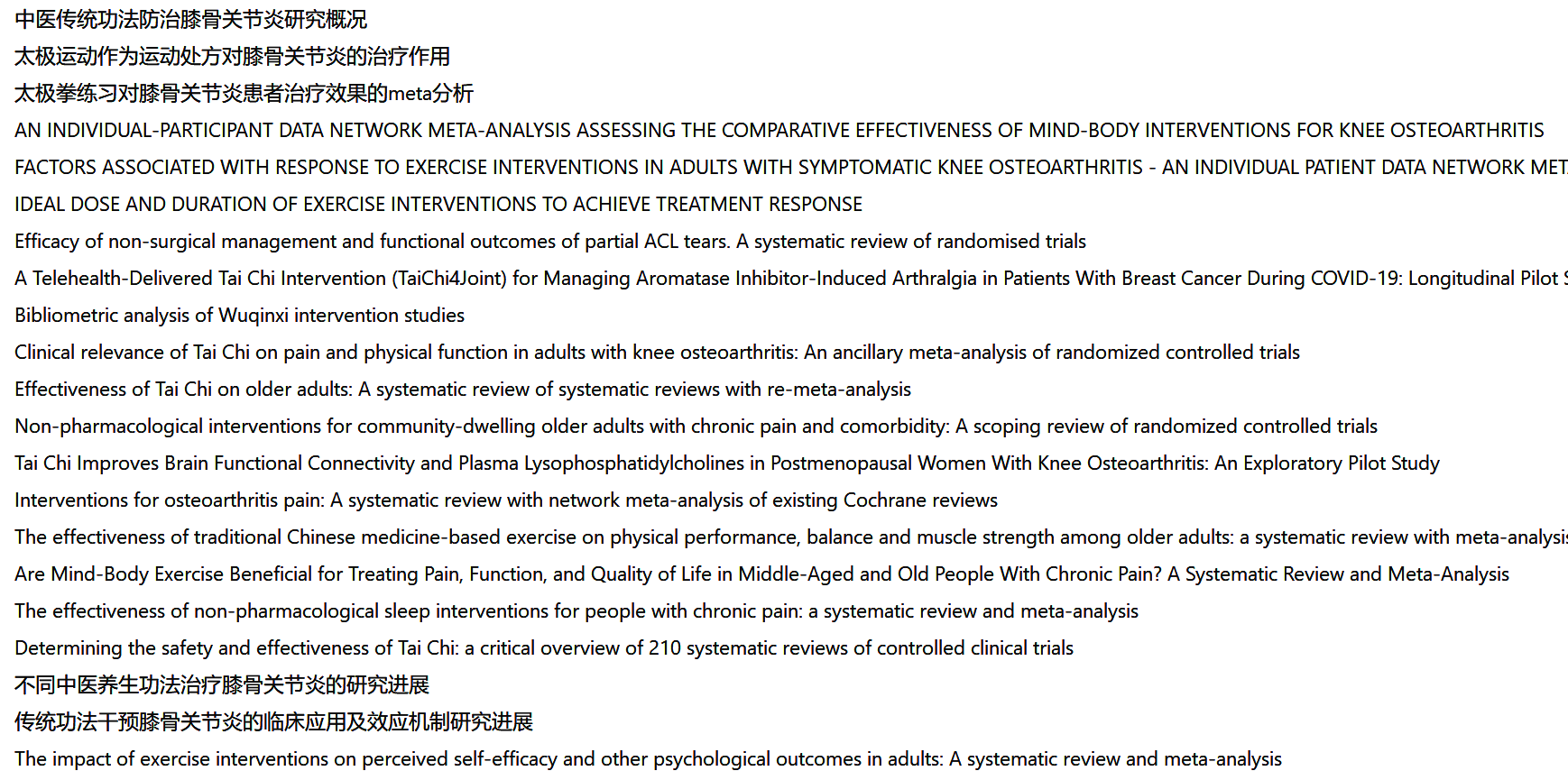

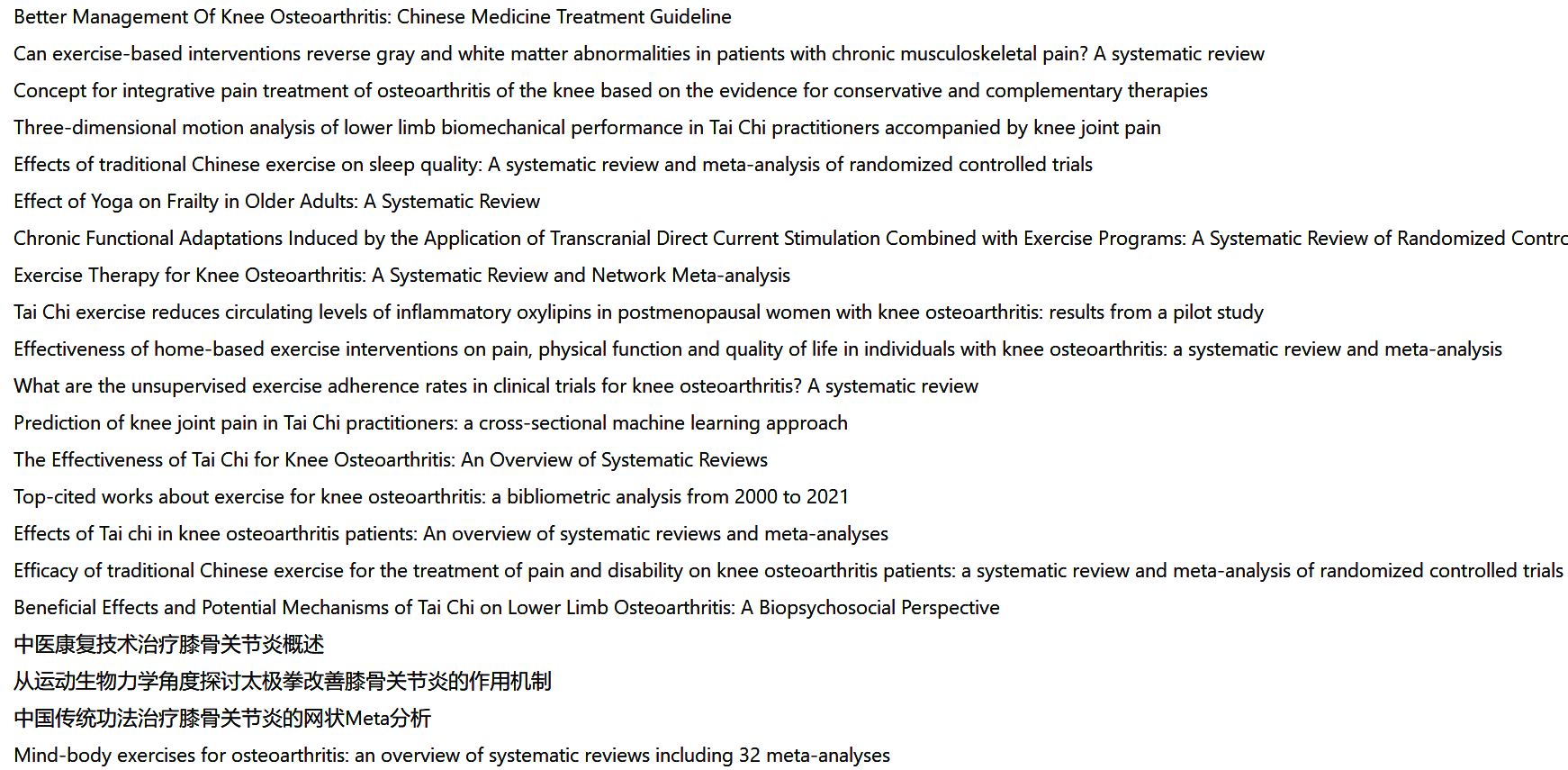

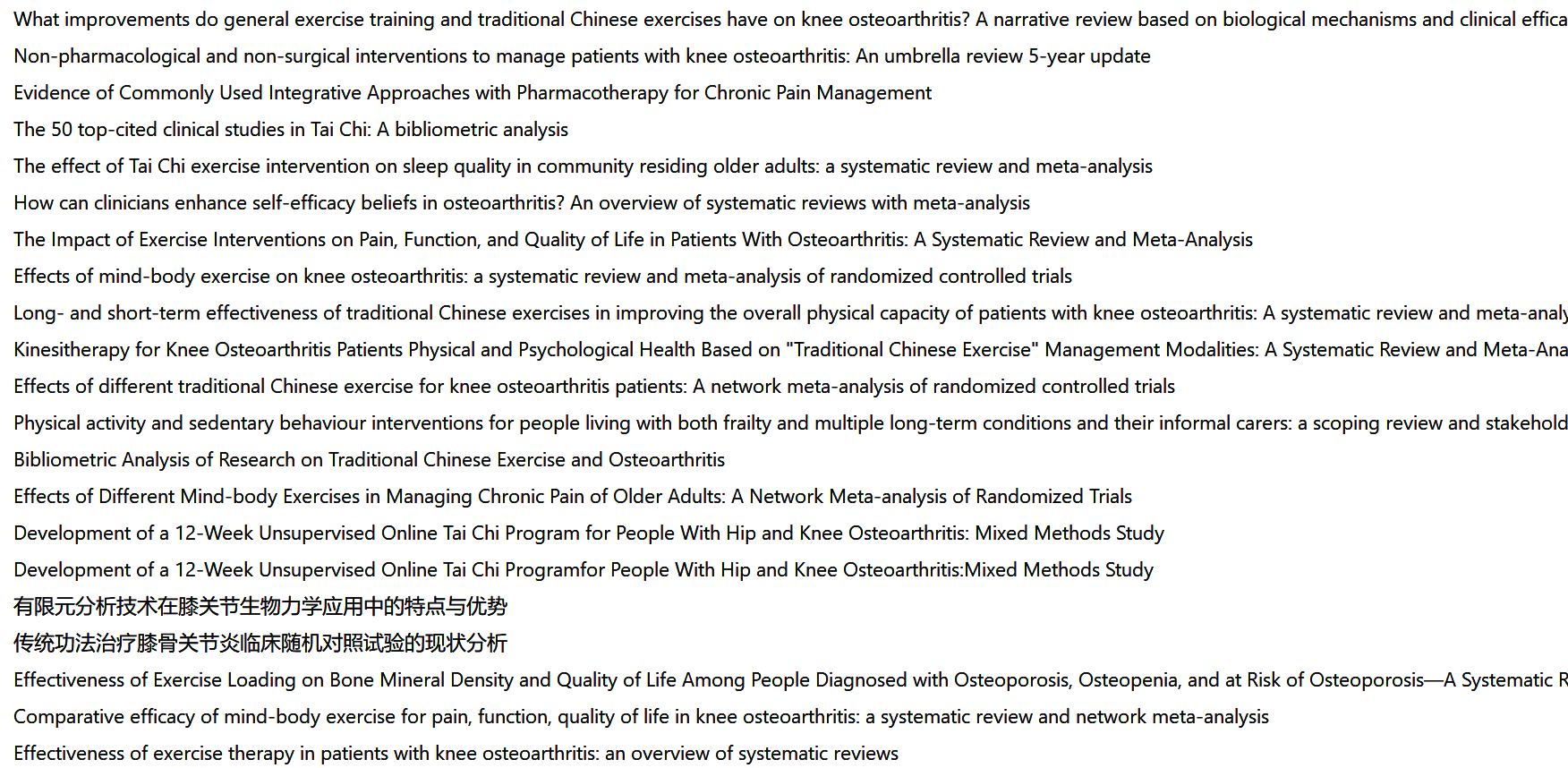

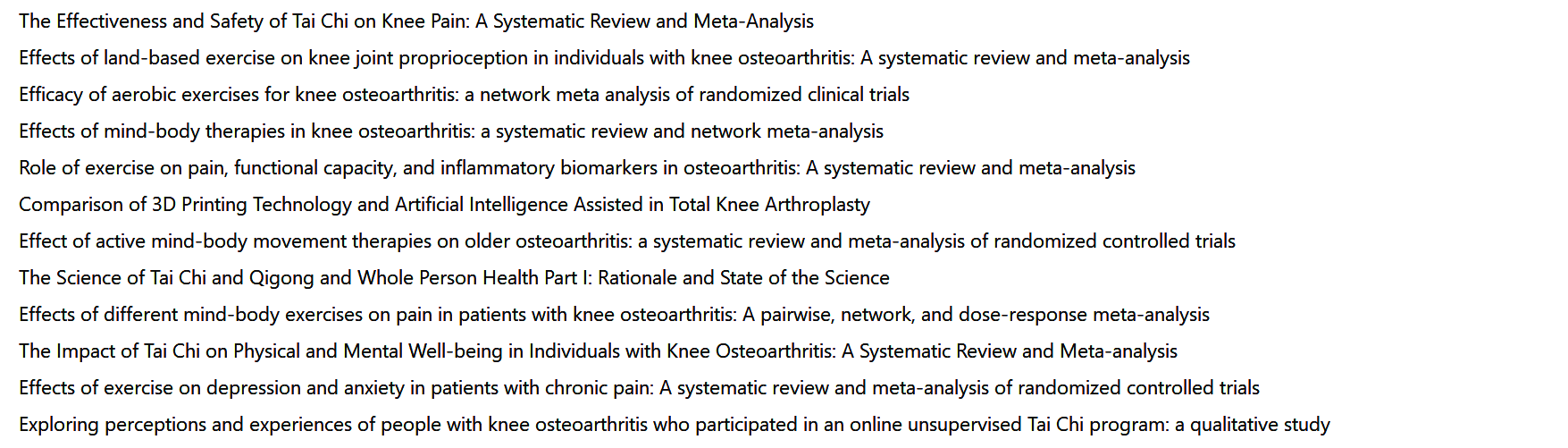


**-Not eligible participants(n=34)**


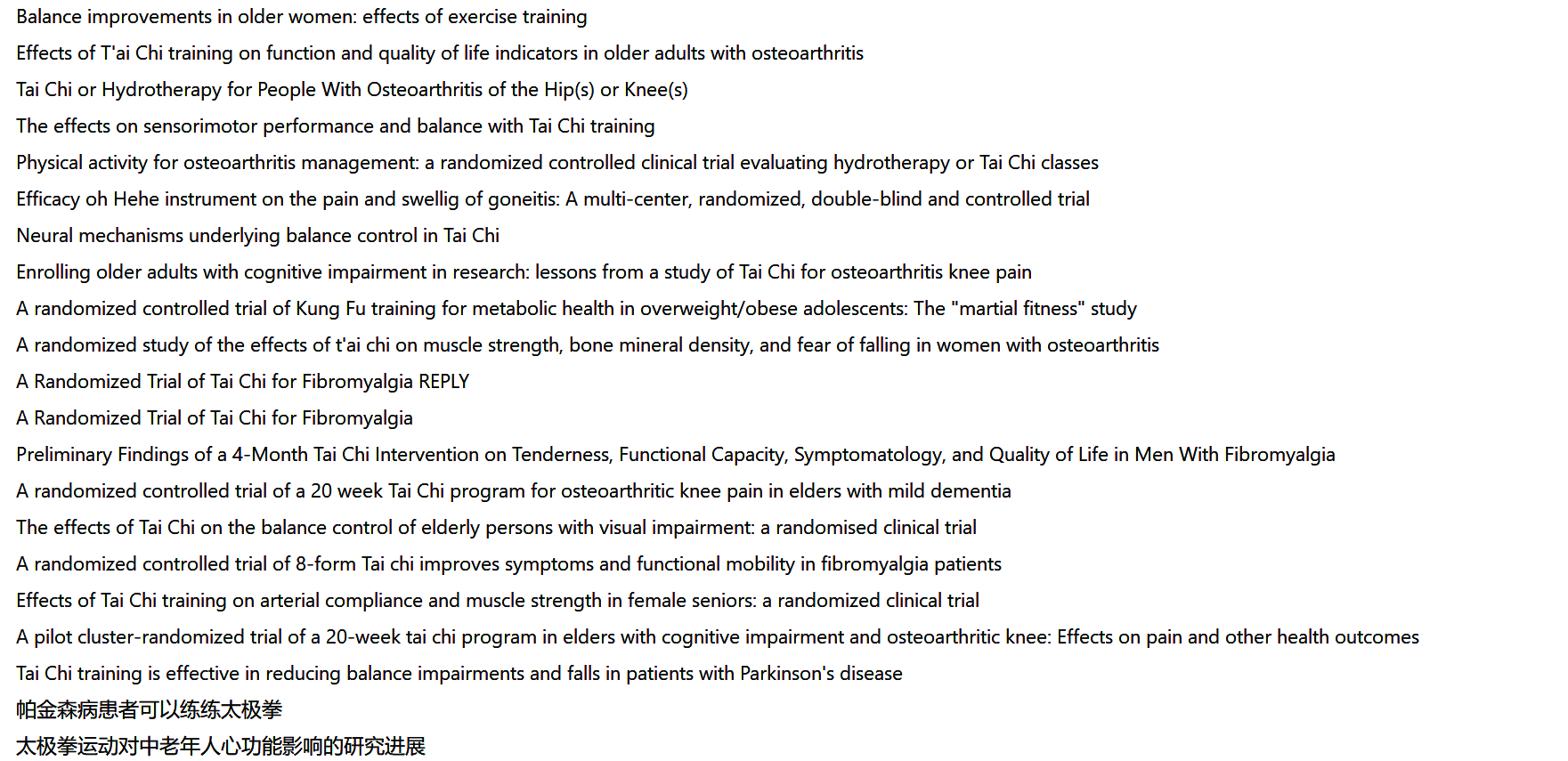

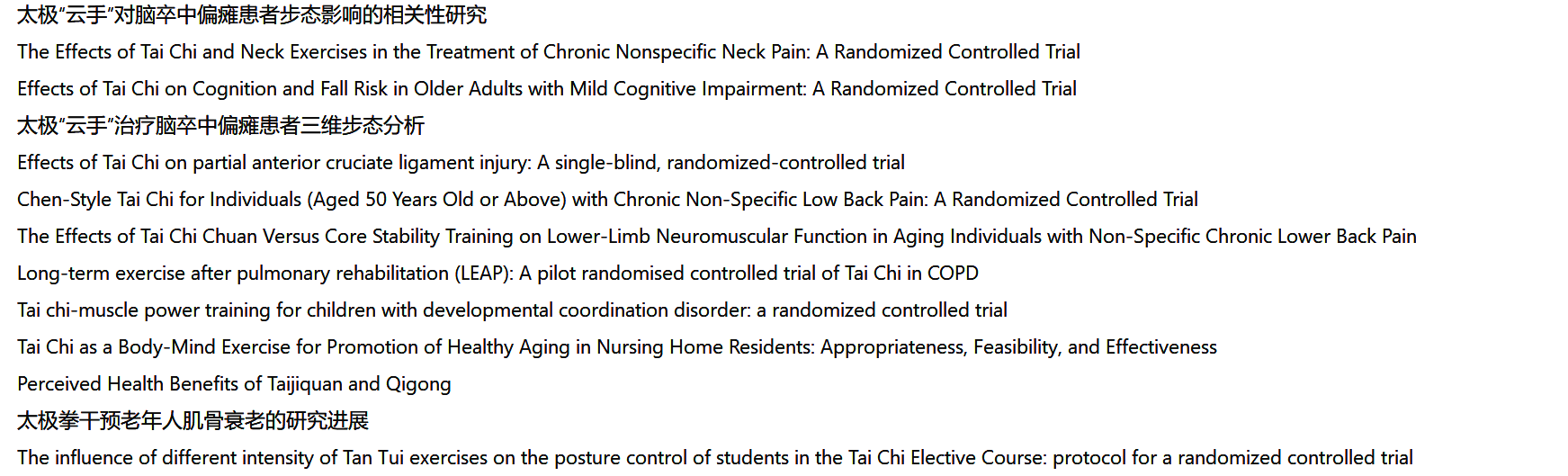


**-Not eligible control group(n=5)**


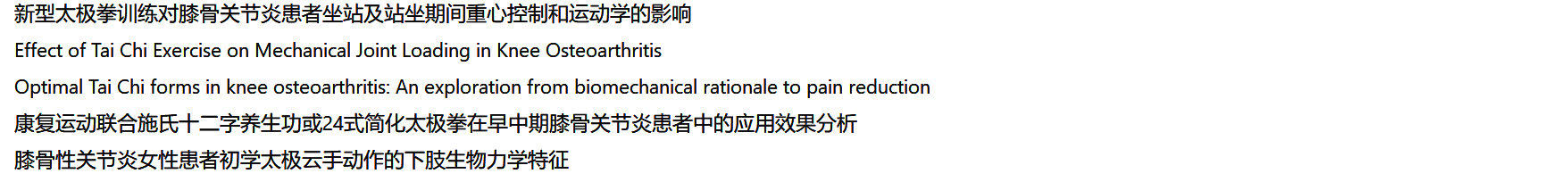


**-Not elgible intervention(n=29)**


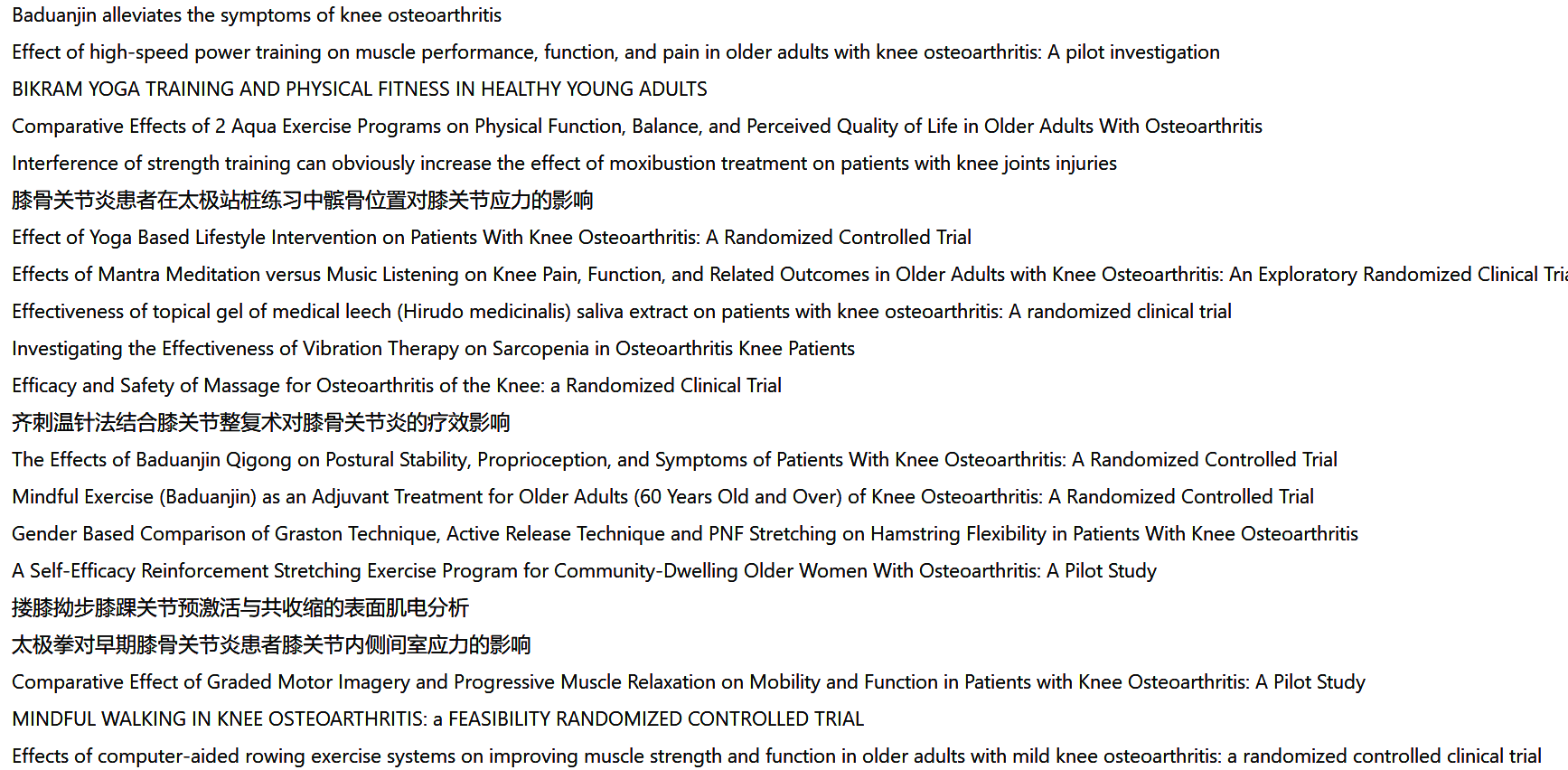

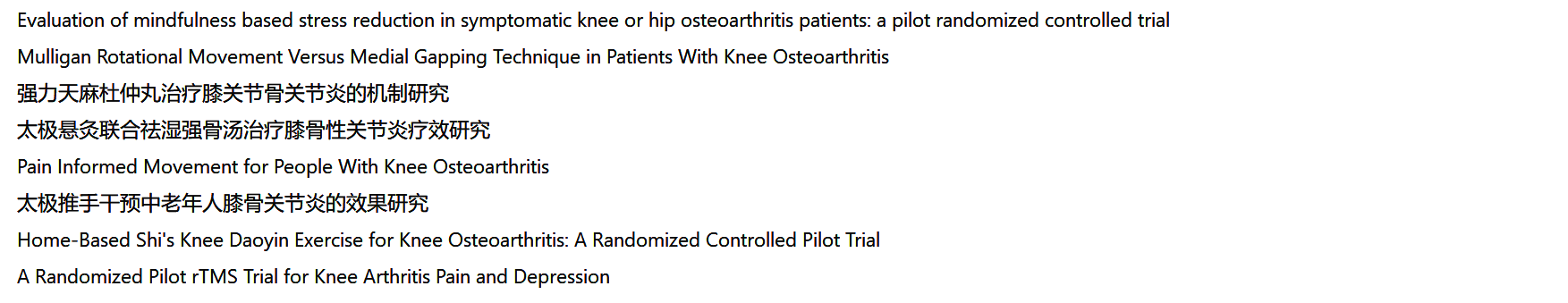


**-Protocol(n=16)**


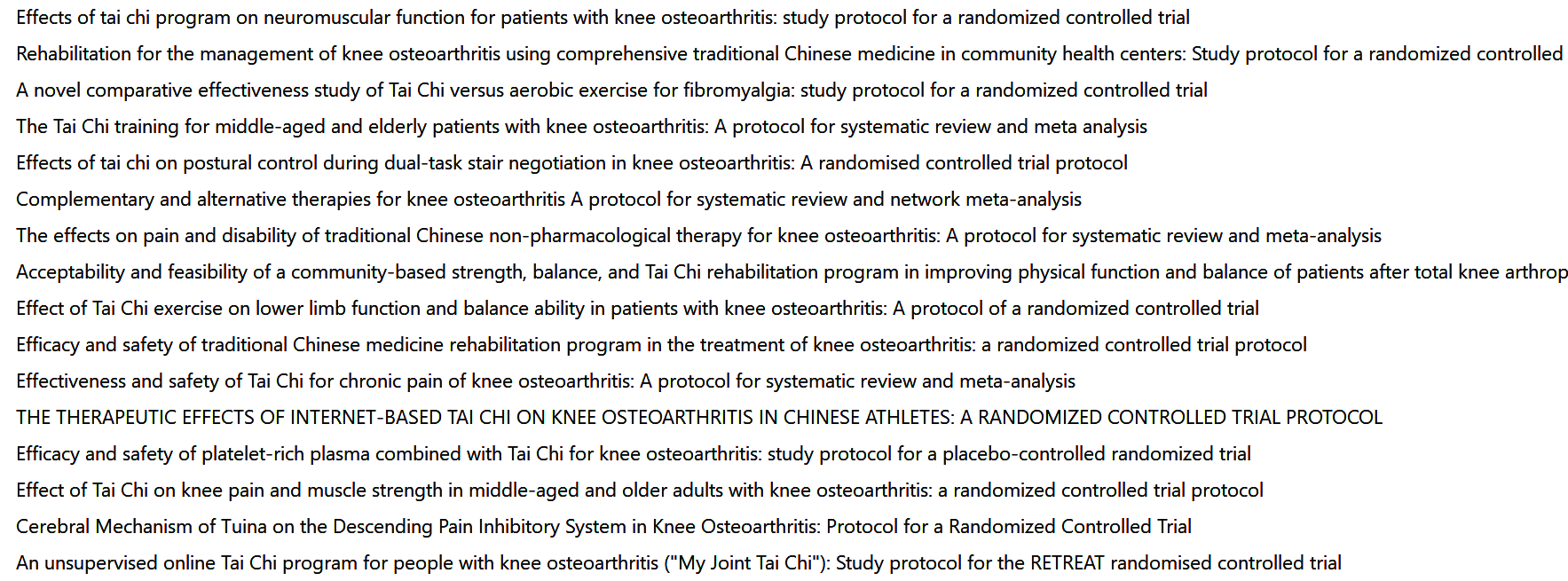


**-Combined therapies(n=6)**


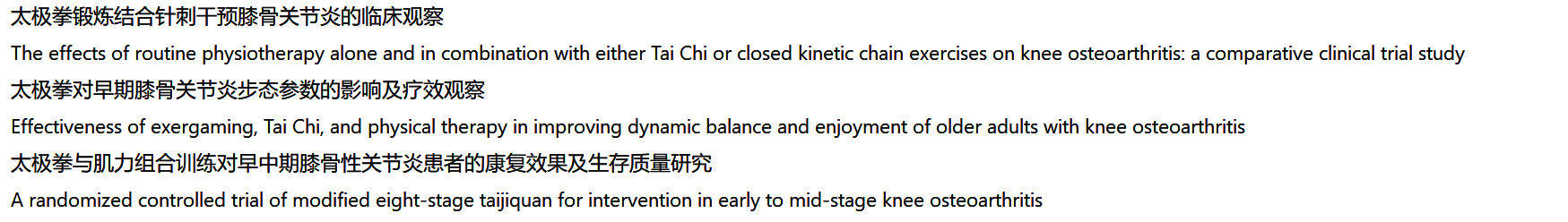


**-postoperative rehabilitation（n=4）**


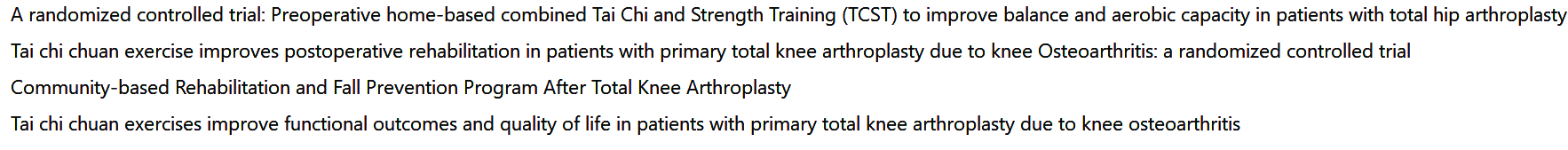


**Full-text  articles  excluded,with reasons(n=35)
-Not  RCT(n=6)**


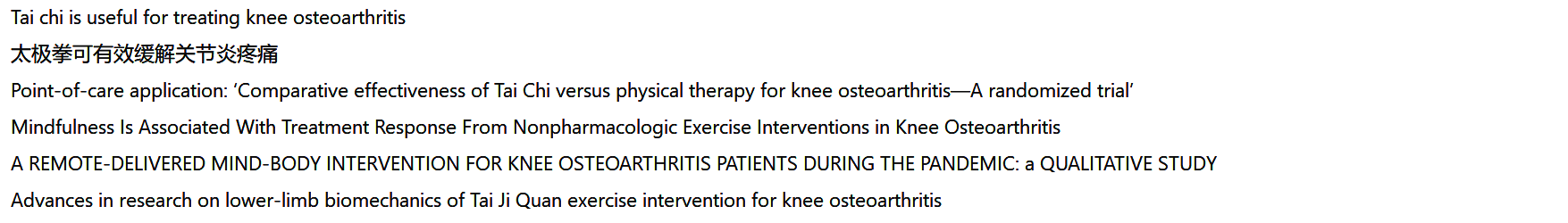


**-Failure to meet outcome indicator（n=8）**


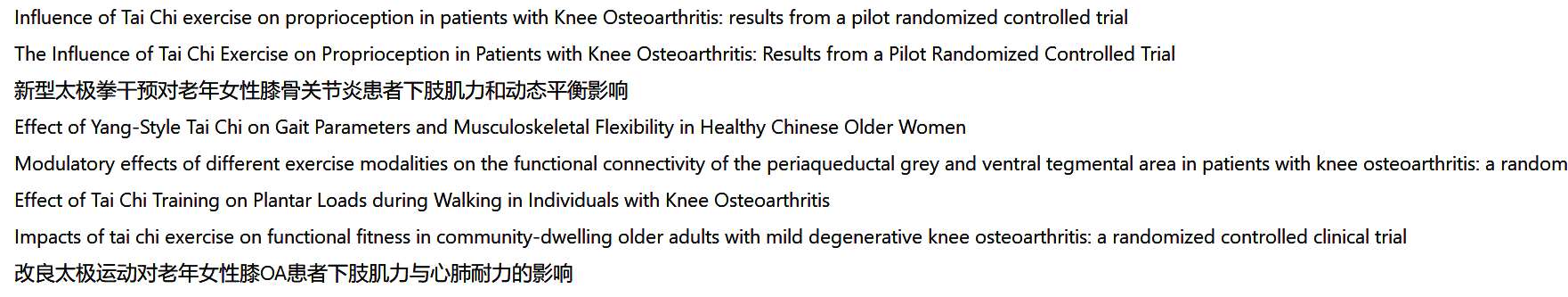


**-Combination therapy(n=3)**


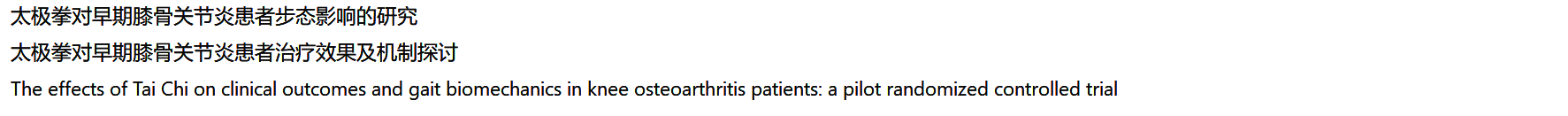


**-Full text not retrieved(n=9）**


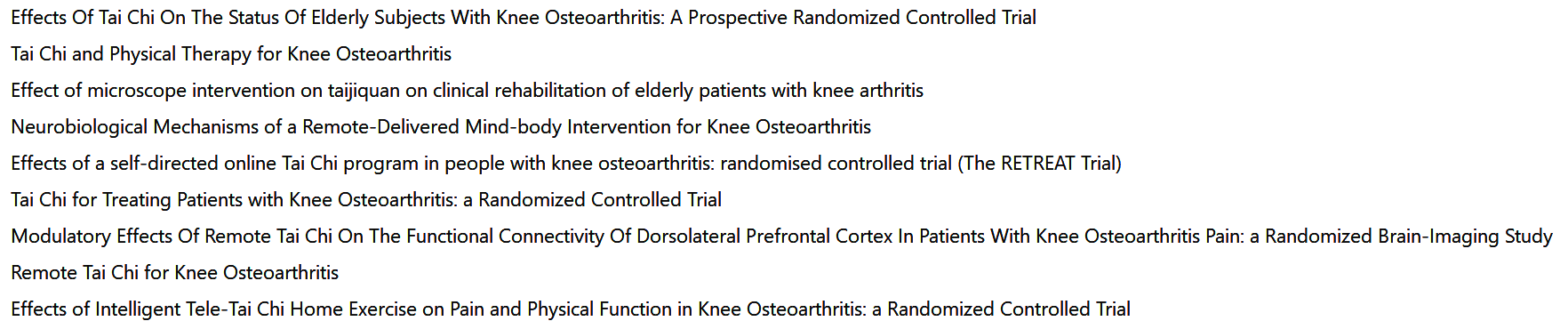


**-Non-English and Chinese(n=1)**


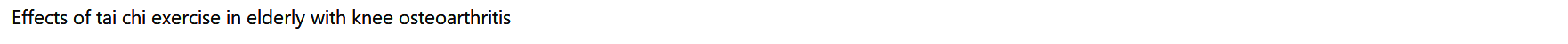


**-Duplicate data(n=7)**


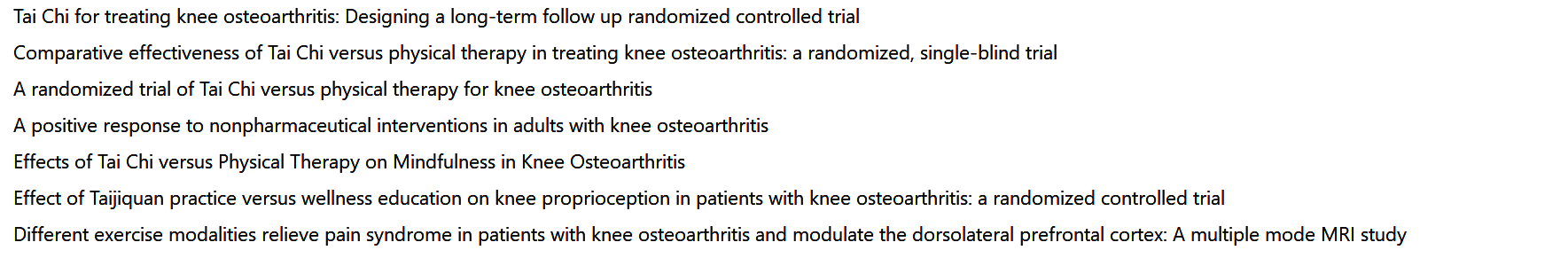


**-Lack of control group(n=1)**


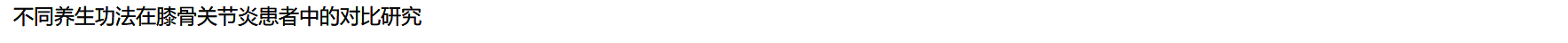


S5 Table. Reasons for risk of bias

| **Reasons for risk of bias** | | | | | | | | | | | | | | | |
| --- | --- | --- | --- | --- | --- | --- | --- | --- | --- | --- | --- | --- | --- | --- | --- |
| **study** | | **Random sequence generation (selection bias)** | | **Allocation concealment (selection bias)** | | **Blinding of participants and personnel (performance bias)** | | **Blinding of outcome assessment (detection bias)** | | **Incomplete outcome data (attrition bias)** | | **Selective reporting (reporting bias)** | | **Other bias** | |
| 1 | peng et al. 2021 | Low risk of bias | Random number table method | Unclear risk of bias | No specific allocation hiding method is mentioned | High risk of bias | The intervention implementers are likely to know the grouping | Unclear risk of bias | It is not clear whether the results were assessed by those who knew about the grouping | Low risk of bias | All patients were treated and evaluated by default | Low risk of bias | The preset outcome was fully reported | Unclear risk of bias | The small sample size may affect the generalizability of the results |
| 2 | Wang et al.2009 | Low risk of bias | Random number table method | Low risk of bias | The randomization protocol was stored in a sealed, opaque envelope | High risk of bias | The intervention implementers are likely to know the grouping | Low risk of bias | Those responsible for the outcome assessment were not aware of the grouping | Low risk of bias | All patients were treated and evaluated by default | Low risk of bias | The preset outcome was fully reported | Unclear risk of bias | The small sample size may affect the generalizability of the results |
| 3 | Zhou et al.2019 | Low risk of bias | random allocation | Unclear risk of bias | No specific allocation hiding method is mentioned | High risk of bias | The intervention implementers are likely to know the grouping | Unclear risk of bias | It is not clear whether the results were assessed by those who knew about the grouping | Low risk of bias | All patients were treated and evaluated by default | Low risk of bias | The preset outcome was fully reported | Unclear risk of bias | The small sample size may affect the generalizability of the results |
| 4 | Wang et al.2016 | Low risk of bias | random allocation | Low risk of bias | The randomization protocol was stored in a sealed, opaque envelope | Low risk of bias | Single-blind evaluation design, intervention standardization and application of objective indicators | Low risk of bias | The assessor blind method was clearly implemented | Low risk of bias | All patients were treated and evaluated by default | Low risk of bias | The preset outcome was fully reported | Low risk of bias | There are no other obvious sources of bias |
| 5 | Kang et al.2022 | Low risk of bias | Random number table method | Unclear risk of bias | No specific allocation hiding method is mentioned | High risk of bias | The intervention implementers are likely to know the grouping | Low risk of bias | The VAS score was recorded by a professional using a standard scale, reducing the risk of measurement bias | Low risk of bias | The number and reasons for the loss of follow-up were reported | Low risk of bias | The preset outcome was fully reported | Unclear risk of bias | The small sample size may affect the generalizability of the results |
| 6 | Lü et al.2017A / Zhu et al.2016B | Low risk of bias | random allocation | Unclear risk of bias | No specific allocation hiding method is mentioned | Low risk of bias | Single-blind evaluation design, intervention standardization and application of objective indicators | Low risk of bias | The assessor blind method was clearly implemented | Low risk of bias | The number and reasons for the loss of follow-up were reported | Low risk of bias | The preset outcome was fully reported | Unclear risk of bias | The small sample size may affect the generalizability of the results |
| 7 | Brisme´e et al.2007 | Low risk of bias | random allocation | Unclear risk of bias | No specific allocation hiding method is mentioned | Low risk of bias | Single-blind evaluation design, intervention standardization and application of objective indicators | Low risk of bias | Those responsible for the outcome assessment were not aware of the grouping | Low risk of bias | The number and reasons for the loss of follow-up were reported | Low risk of bias | The preset outcome was fully reported | Unclear risk of bias | The small sample size may affect the generalizability of the results |
| 8 | Song et al.2003 | Low risk of bias | random allocation | Unclear risk of bias | No specific allocation hiding method is mentioned | High risk of bias | Inadequate intervention standardization | Low risk of bias | It was performed by exercise physiologists using a blind method | Low risk of bias | The number and reasons for the loss of follow-up were reported | Low risk of bias | The preset outcome was fully reported | Unclear risk of bias | The small sample size may affect the generalizability of the results |
| 9 | Hu et al.2019 | Low risk of bias | random allocation | Unclear risk of bias | No specific allocation hiding method is mentioned | Low risk of bias | Single-blind evaluation design, intervention standardization and application of objective indicators | Low risk of bias | The assessor blind method was clearly implemented | Low risk of bias | The number and reasons for the loss of follow-up were reported | Low risk of bias | The preset outcome was fully reported | Low risk of bias | There are no other obvious sources of bias |
| 10 | Song et al.2022 | Low risk of bias | Random number table method | Low risk of bias | The randomization protocol was stored in a sealed, opaque envelope | Low risk of bias | Single-blind evaluation design, intervention standardization and application of objective indicators | Low risk of bias | Those responsible for the outcome assessment were not aware of the grouping | Low risk of bias | All patients were treated and evaluated by default | Low risk of bias | The preset outcome was fully reported | Unclear risk of bias | The small sample size may affect the generalizability of the results |
| 11 | Michael et al.2013 | Low risk of bias | random allocation | Unclear risk of bias | No specific allocation hiding method is mentioned | Unclear risk of bias | Not specified | Unclear risk of bias | It is not clear whether the results were assessed by those who knew about the grouping | Low risk of bias | The number for the loss of follow-up were reported | Low risk of bias | The preset outcome was fully reported | Unclear risk of bias | The small sample size may affect the generalizability of the results |
| 12 | Lee et al.2009 | Low risk of bias | random allocation | Unclear risk of bias | No specific allocation hiding method is mentioned | High risk of bias | The intervention implementers are likely to know the grouping | Low risk of bias | The assessor blind method was clearly implemented | Low risk of bias | The number and reasons for the loss of follow-up were reported | Low risk of bias | The preset outcome was fully reported | Unclear risk of bias | The small sample size may affect the generalizability of the results |
| 13 | Zhang et al.2025 | Low risk of bias | Random number table method | Unclear risk of bias | No specific allocation hiding method is mentioned | Unclear risk of bias | Not specified | Low risk of bias | The assessor blind method was clearly implemented | Low risk of bias | The number and reasons for the loss of follow-up were reported | Low risk of bias | The preset outcome was fully reported | Unclear risk of bias | The small sample size may affect the generalizability of the results |

S6 Table. Egger’s test for each outcome

**WOMAC Pain**


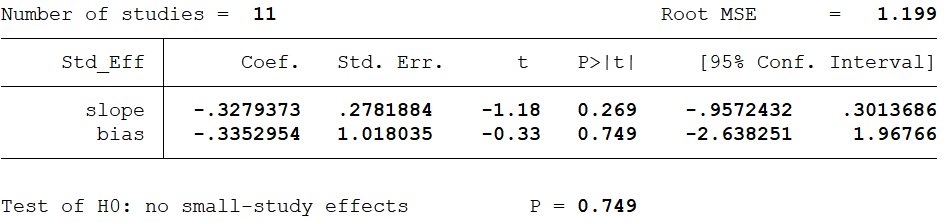


**WOMAC Stiffness
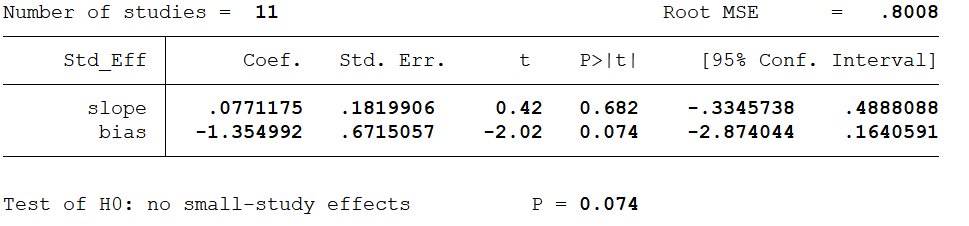
**

**WOMAC Function**


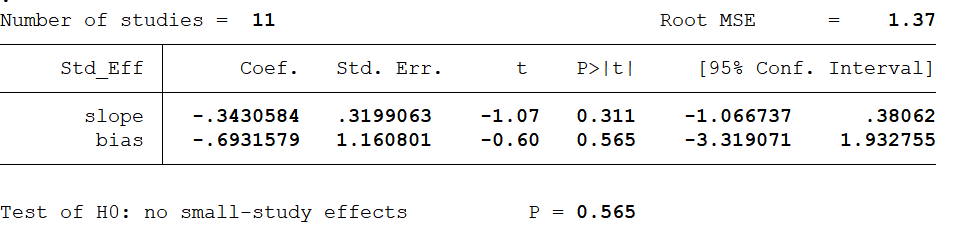


S1 Figure. Subgroup analysis of the effect of Tai Chi on WOMAC pain/WOMAC stiffness/WOMAC physical function/VAS pain/SF-36 PCS/SF-36 MCS in patients with KOA

**WOMAC pain**


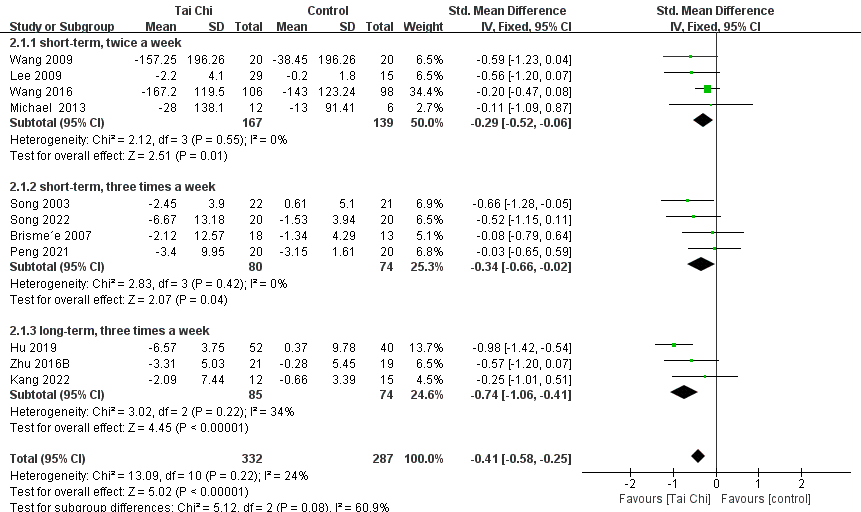


**WOMAC stiffness**


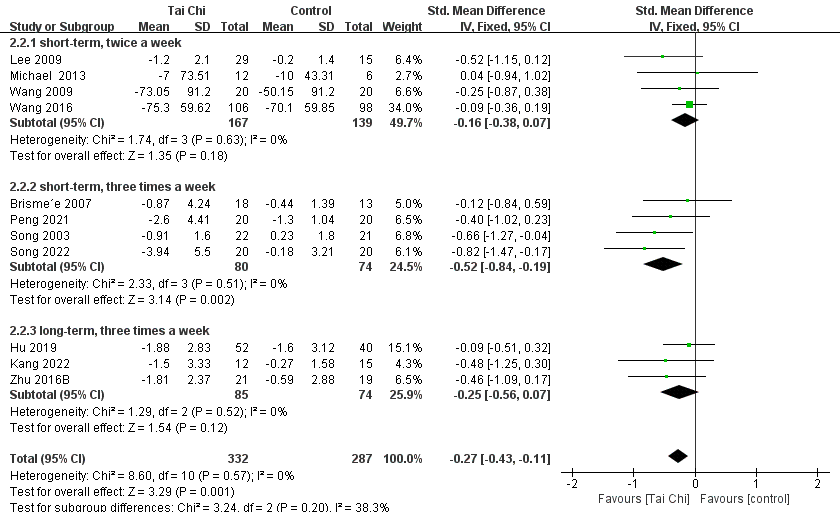


**WOMAC physical function**


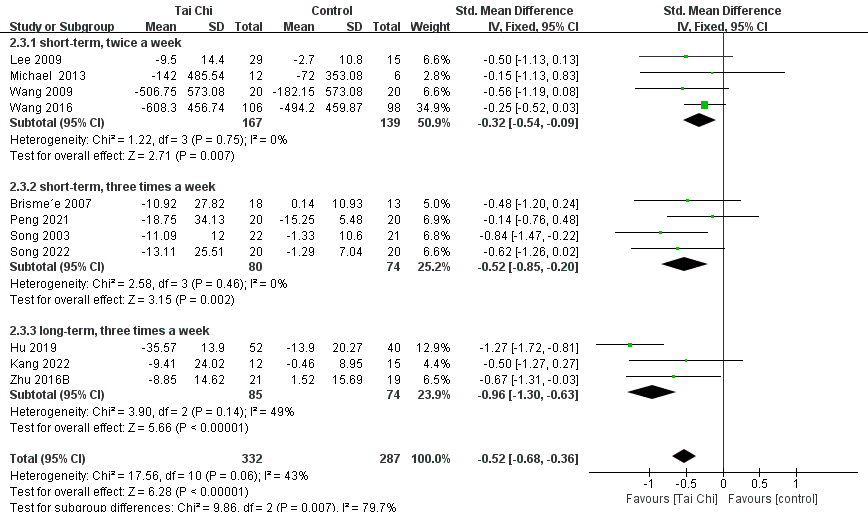


**VAS pain**


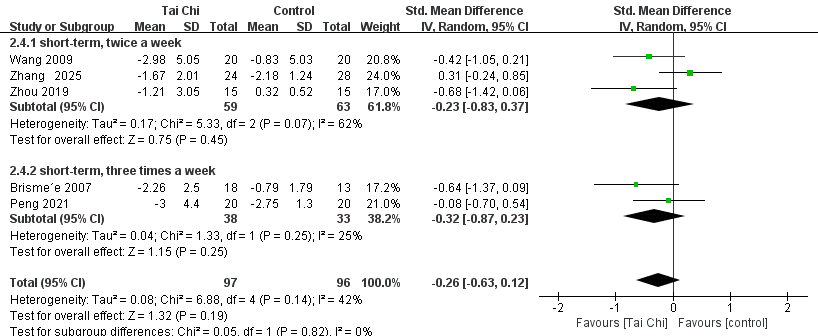


**SF-36 PCS**


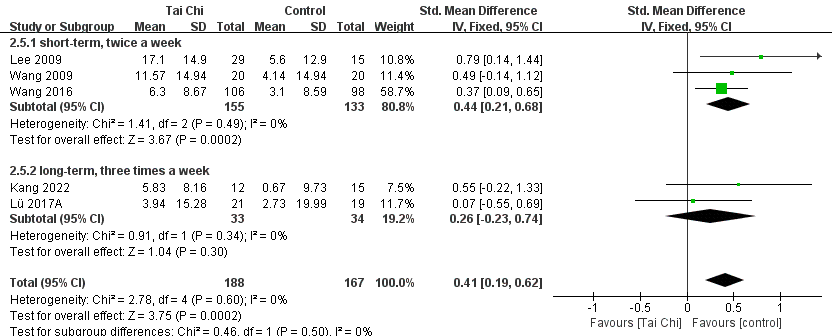


**SF-36 MCS**


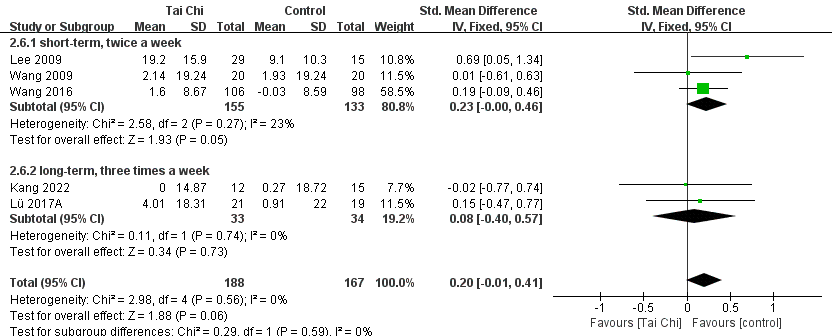


**Yang-style Tai Chi WOMAC pain**


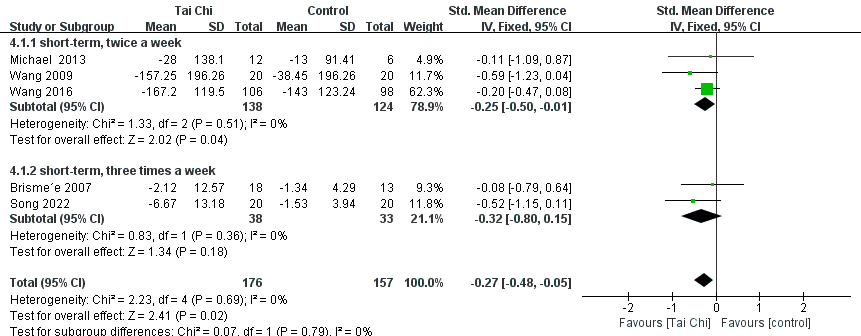


**Yang-style Tai Chi WOMAC stiffness**


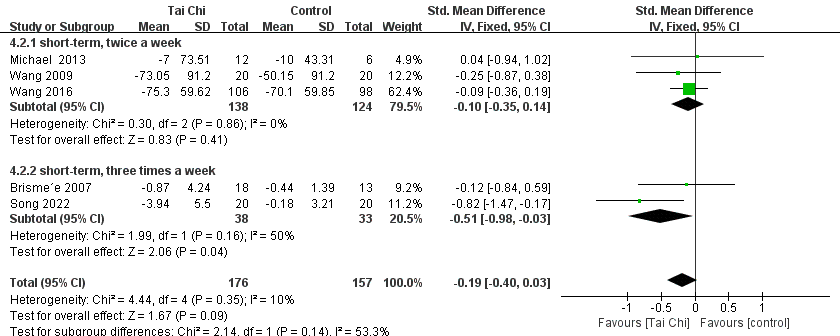


**Yang-style Tai Chi WOMAC function**


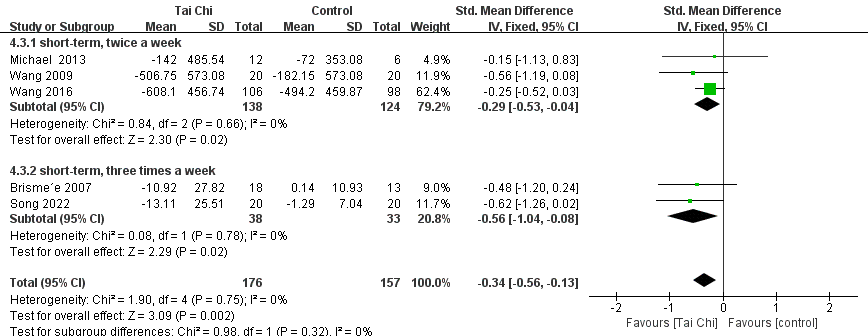


S2 Figure. Sensitivity Analysis

**WOMAC pain**

**WOMAC stiffness**

**WOMAC physical function**

**VAS pain**

**SF-36 PCS**

**SF-36 MCS**

S3 Figure. Heterogeneity after excluding Song (2022)


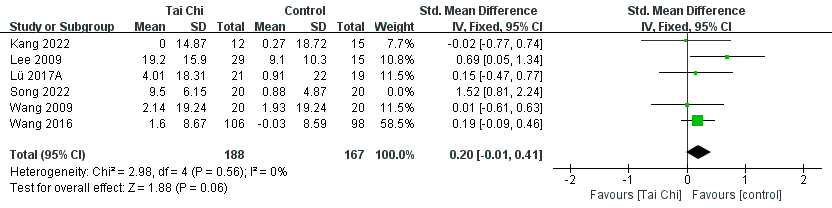


**S4 Figure. Heterogeneity after excluding Zhang（2025）**


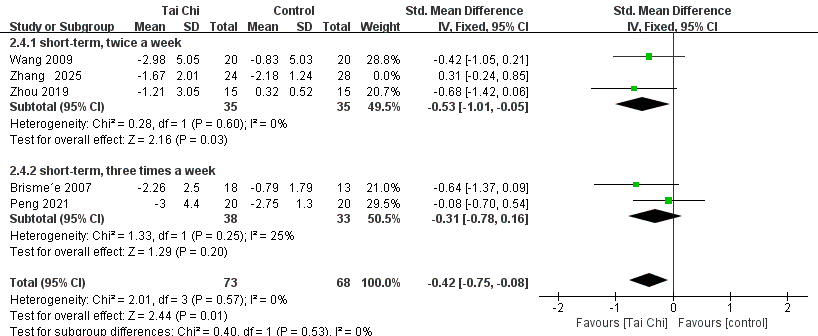

Supplement: Supplementary file 1 [file Table_1.docx]
